# Thermal Mapping of Self-Promoted Calcium Carbide Reactions for Performing Energy-Economic Processes

**DOI:** 10.3390/ijms23052763

**Published:** 2022-03-02

**Authors:** Konstantin S. Rodygin, Kristina A. Lotsman, Kirill S. Erokhin, Viktoria A. Korabelnikova, Valentine P. Ananikov

**Affiliations:** 1 Institute of Chemistry, Saint Petersburg State University, Universitetskiy pr. 26, 198504 Saint Petersburg, Russia; k.rodygin@spbu.ru (K.S.R.); k.lotsman@spbu.ru (K.A.L.); 2 Zelinsky Institute of Organic Chemistry, Russian Academy of Sciences, Leninsky pr. 47, 119991 Moscow, Russia; erokhin@ioc.ac.ru (K.S.E.); korabelnikova@ioc.ac.ru (V.A.K.)

**Keywords:** molecular reactions, thermal mapping, energy economy, energy saving, calcium carbide, acetylene, 3D printing

## Abstract

The syntheses of various chemical compounds require heating. The intrinsic release of heat in exothermic processes is a valuable heat source that is not effectively used in many reactions. In this work, we assessed the released heat during the hydrolysis of an energy-rich compound, calcium carbide, and explored the possibility of its usage. Temperature profiles of carbide hydrolysis were recorded, and it was found that the heat release depended on the cosolvent and water/solvent ratio. Thus, the release of heat can be controlled and adjusted. To monitor the released heat, a special tube-in-tube reactor was assembled using joining part 3D-printed with nylon. The thermal effect of the reaction was estimated using a thermoimaging IR monitor. It was found that the kinetics of heat release are different when using mixtures of water with different solvents, and the maximum achievable temperature depends on the type of solvent and the amount of water and carbide. The possibility of using the heat released during carbide hydrolysis to initiate a chemical reaction was tested using a hydrothiolation reaction—the nucleophilic addition of thiols to acetylene. In a model experiment, the yield of the desired product with the use of heat from carbide hydrolysis was 89%, compared to 30% in this intrinsic heating, which was neglected.

## 1. Introduction

Chemical reactions may proceed with heat release (exothermic), with heat consumption (endothermic), or nearly thermoneutral [1,2]. In the first case, which is typical for many synthetic targets, additional heat is required.

At a certain stage, some amount of heat may be released upon a reaction of energy-rich components of the reaction. In regular chemical practice, rather often, this heat remains unused or even requires extra energy for freezing. It is a very common case to supply or remove heat at each step individually, thus resulting in double power consumption (i.e., power for freezing in one stage and heating in another stage). Indeed, the design of energy-economic processes is scarcely developed.

Many attempts have been made to combine exo- and endothermic processes. As a result, autothermal reaction setups were developed [3]. However, the scope of autothermal reactions is limited. The extra heat in these processes is consumed by reactions taking place in the same media or by a limited number of other compatible reactions. For multistep organic synthesis procedures, applying an energy-economic concept is not a common practice.

**Scheme 1:**
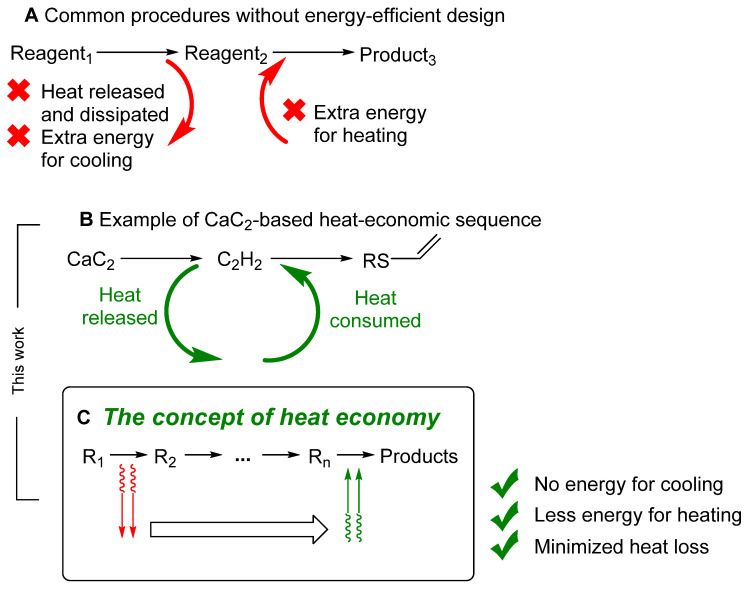
Heat transfer and heat consumption strategy in reaction design: (**A**)—heat supply and heat consumption are not combined; (**B**)—heat supply and heat consumption are combined on the example of calcium carbide; (**C**)—the concept of a heat economy.

To show an illustration, exothermic reactions provide heat, which usually dissipates into the environment or is removed using freezing devices (Scheme 1A). In the subsequent step, heating may be required (Scheme 1A). Using the heat from the first step in the second step is a valuable option in step-by-step transformations. Furthermore, an exothermic reaction can be used as a heat source for an endothermic reaction, and heat transfer from one reaction to another can be implemented both in sequential and individual organic transformations. Thus, the heat of one reaction can be used to perform even a completely unrelated reaction. This option allows us to direct the heat from the first reaction to the heating required for another reaction and at the same time does not waste additional energy for cooling for the first process.

In the present study, the possibility and impact were checked on the nucleophilic addition of thiols to acetylene generated in situ from calcium carbide (Scheme 1B). The reaction includes two steps: hydrolysis of calcium carbide (as an exothermic reaction), which provides extra heat and needs to be cooled (otherwise, acetylene polymerization occurs). The second step is the addition of a thiol to gaseous acetylene, which proceeds efficiently only when heated. Thus, applying the heat released while an exothermic reaction occurs (hydrolysis of calcium carbide) as a heat source for an endothermic reaction (thiol addition to acetylene) is a good opportunity to implement a heat-economic process. It is important to note a chemical connection between the stages: acetylene is produced in the first stage and consumed in the second stage.

In this work, the effect of heat generated was estimated, and the possibility of using it to promote a chemical transformation was identified. Calcium carbide was used as a model energy-rich compound. Calcium carbide hydrolysis is an exothermic process accompanied by the release of a significant amount of energy in the form of heat. The enthalpy of the reaction is −127.2 kJ/mol (30 kcal/mol) [4]. In fact, 1 ton of calcium carbide can provide 469 Mcal of energy. This amount of thermal energy should be withdrawn from the reaction mixture; otherwise, the forming acetylene will polymerize or may even explode. According to stoichiometry, the hydrolysis of 1 kg of carbide requires only 0.56 L of water. Under industrial manufacturing conditions, 5 to 20 L of water is used to cool the reaction mixture and prevent polymerization or explosion. Recently, calcium carbide was actively used in the construction of heterocycles [5,6,7,8,9,10,11,12,13], vinylation processes [14,15], synthesis of monomers [16,17], mechanochemical processes [18,19,20,21,22], and many other organic transformations [23,24,25]. The key advantage of calcium carbide is its renewable potential [26]. If heat can be used for the promotion of chemical reactions, the use of carbides would be more sustainable.

Base- and water-sensitive reactions with calcium carbide may be performed in a special 3D printed reactor, and the acetylene generation part is separated from the reaction part [27]. However, in this case, the heat of the hydrolysis of the carbide is lost due to the separation of the chambers.

**Figure 1:**
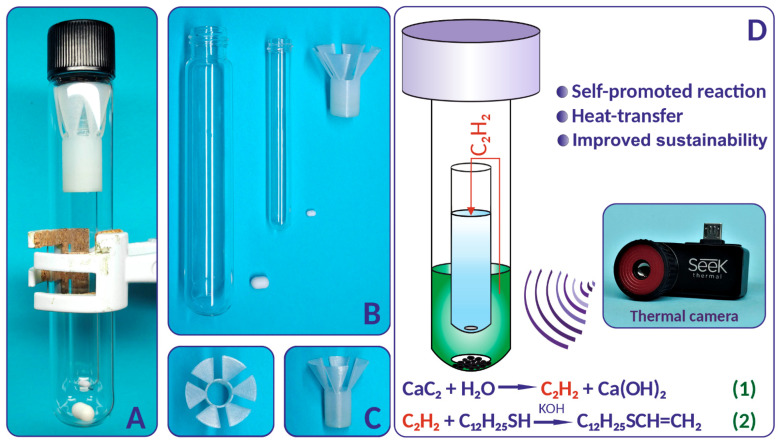
The reaction setup using the developed tube-in-tube reactor: (**A**)—the reactor ready to use; (**B**)—the disassembled reactor; (**C**)—nylon liner; (**D**)—thermal camera and the scheme of tube-in-tube reactor and two stepwise chemical reactions involved.

Here, a tube-in-tube reactor was developed (Figure 1). The reactor consisted of an outer tube and inner tube (Figure 1A, assembled; and B, disassembled) and a nylon liner (Figure 1C). Thus, the calcium carbide in this process acted simultaneously as a supplier of gaseous acetylene and as a supplier of thermal energy. The hydrolysis of calcium carbide occurred in the outer tube, providing heat and acetylene gas (Figure 1, reaction 1). Then, the acetylene moved to the inner tube and was consumed by the reaction mixture containing the thiol and the base (Figure 1, reaction 2).

To clarify the subject for the readers, the attempts to combine exothermic and endothermic processes represent a long-standing aim, and autothermal processes were developed for a long time. The idea of using heat release in one stage to accelerate another stage of a multistep synthesis and to improve energy efficiency is obvious. By no means may the present article claim to describe these issues for the first time.

The novelty and importance of the present study should be pointed out. Concerning novelty, we demonstrate for the first time that, by using cosolvents, it is possible to control the temperature profile to a substantial degree and to achieve the best heat release–consumption combination. An application was demonstrated for the thiols vinylation reaction to produce a useful monomer from calcium carbide. It should be emphasized that energy-economic processes are currently not actively implemented in research projects, and, here, we want to promote a broad discussion of this important subject.

## 2. Results and Discussion

### 2.1. Design of the Tube-In-Tube Reactor

We developed a special type of tube-in-tube reactor. A small tube was placed inside a larger screw-capped tube. The smaller tube was kept inside of a large tube by means of a special liner digitally designed and printed on a 3D printer. Thus, the tubes did not come into contact with each other, which made it possible to separate the reagents of two different reactions, avoiding mixing them, and, at the same time, the gas phase in both test tubes was shared (for acetylene gas transfer). The reactor was made of commercially available tubes and was simple to use, did not require glass-blowing work, and was easy to assemble and disassemble. Nylon was chosen as the material for the 3D printing of a liner due to its resistance to most organic solvents [27] and stability at high temperatures.

#### 3D Printing of the Nylon Liner

3D printing is a powerful and flexible tool in various applications to generate specific and unique units [28,29,30,31,32]. To perform the reaction in a tube-in-tube reactor, it was necessary to placed one tube inside the other without contacting the walls and, especially, the bottom. At the same time, the ability to remove the inner tube from the outer tube for unloading products should be preserved. Additionally, the liner should be stable under high temperatures and resistant to organic solvent vapors. These requirements are met by nylon, which was tested in the present study. Several liner designs were developed and optimized (please see Supplementary Material, Section S1, Figure S1). Due to the precisely selected diameter, the liner was placed on and sat tightly on the inner tube, and the expanding petals held the small tube inside the large tube. After the reaction, the small tube can easily be removed with pincers by pulling it up, and then the liner can also be removed when the inner tube is already removed. Thus, the liner can be used several times. When carrying out reactions with large loads, two liners can be applied. In addition, the liner dimensions are easily scalable and can be matched to the exact size of small and large tubes. A description of the 3D printing and an adjustable digital model as an .stl file are provided in the Supporting Information.

### 2.2. Thiovinylation Reaction Profile

The vinylation reaction of acetylene with dodecanethiol was chosen as a model reaction to estimate the heat effect of carbide hydrolysis. The thiol-yne reaction is a well-known process in which a thiol is added to the acetylene gas released from carbide, resulting in a thiovinyl ether. The yield of ether reflects the effect of temperature since heating significantly increases the yield of ether. At the same time, the reaction does not proceed easily, and prolonged and intense heating is required to achieve quantitative yields. The product yield can also easily be checked with NMR, where the vinyl ether signals are clearly separated from those of the starting thiol. Thus, calcium carbide and a solvent were placed in the outer tube, and the thiol, solvent, and base were loaded into the inner tube. Furthermore, a certain amount of water was added into an external tube with carbide along the wall, stirring was switched on, the effect of the released heat on the temperature was recorded with a thermal imager, and the amount of ether produced was estimated by ^1^H NMR.

**Figure 2:**
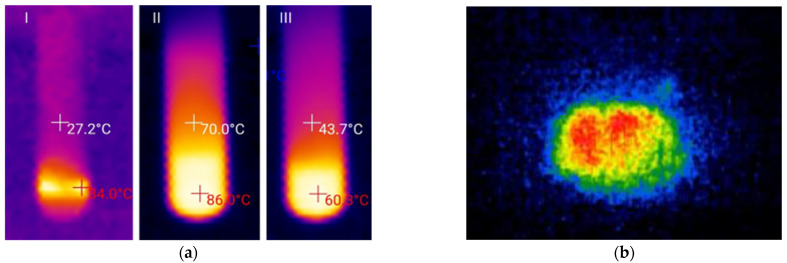
(**a**) Thermal mapping of the reaction of carbide with water according to thermal imager data. I: adding water to DMSO; II: hydrolysis of calcium carbide and acetylene generation; III: cooling after carbide hydrolysis; (**b**) hydrolysis of one granule of calcium carbide under magnification.

To assess the thermal effect online, a thermal imager was installed, and measuring data at selected time periods resulted in heat curves. The first experiments were very promising: the imager precisely fixed each addition of water and each mixing operation. The heating zones and the progress of heat release were clearly seen inside of the tube-in-tube reactor (Figure 2a). The heat released during the hydrolysis of carbide was detected and mapped over time using an IR monitor.

Control experiments were carried out to check the reliability of this setup and the influence of the solvent and the walls of the vessel. Calcium carbide granules were placed on glass, and a DMSO/water mixture was added. A camera was connected to the IR monitor, in front of which a magnifying glass was installed. As a result, an individual granule of calcium carbide was identified, and the heating progress was recorded (Figure 2b). Thus, heating occurred directly on the surface of the granules, and then the heat spread throughout the entire system.

**Figure 3:**
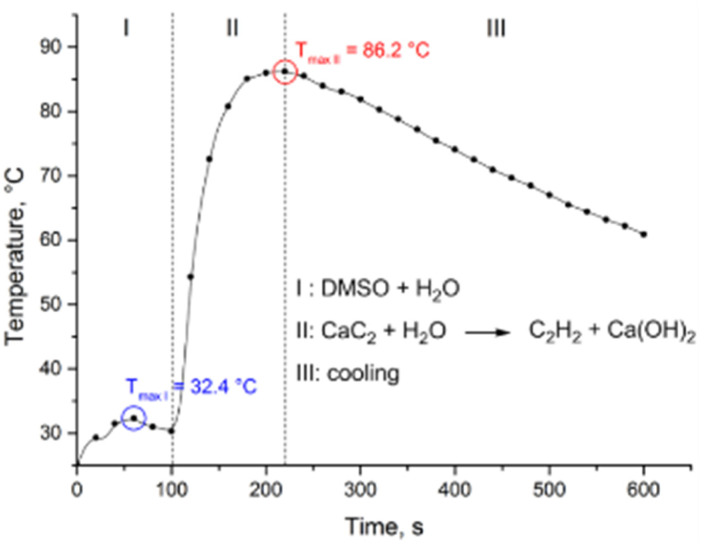
Thermal imaging of the hydrolysis of carbide: (I) the effect of water addition to DMSO; (II) the maximum temperature of the reaction; (III) the cooling stage. The reaction conditions were DMSO (4 mL), H_2_O (2 mL), and CaC_2_ (1.76 g).

The thermal imager automatically showed the minimal and maximal temperatures and heat progress and recorded the data. Initially, there was mild heating due to the mixing of DMSO and water (Figure 2a, I). After turning on the stirring, the carbide was rapidly hydrolyzed with water, and the temperature of the mixture increased from 32–34 to 86–90 °C (Figure 2a, II). Then, the temperature of the mixture slowly decreased (Figure 2a, III) due to heat transfer and consumption. The temperature profile of the same reaction with the same progress was recorded (Figure 3).

The reaction temperature profile consisted of three zones. The first zone was accompanied by slight heating caused by the addition of water to DMSO. The imager was sensitive enough to record the thermal effect of the addition of water to DMSO. Most likely, the formation of hydrogen bonds between the water and DMSO molecules was accompanied by the release of heat. When adding water to DMSO, stirring was turned off, so this is precisely the effect of hydrogen bonding and not partial hydrolysis of the carbide. Furthermore, after intensive stirring was turned on, the carbide reacted vigorously with water, which was accompanied by a rapid release of heat. During the third stage, cooling of the reactor was observed. The rate of cooling depended on the thermal insulation of the reactor and room temperature.

Control experiments were also carried out: a reaction tube was filled with DMSO, a regular contact thermometer was placed into the solvent (not the IR monitor), and an appropriate amount of water was dropped to DMSO in the absence of reagents. Increasing temperature was detected, and the results were the same as in the case of a tube-in-tube reactor (see Supplementary Material, Section S8). Thus, the initial increase in temperature in the reaction mixtures was caused by DMSO–water interactions (e.g., Figure 3, T_max_ = 32.4 °C).

### 2.3. Optimization of the Heat Release Profile

Of course, the total heat that should be released during the complete hydrolysis of calcium carbide is a constant value for the same amount of calcium carbide. However, the kinetics of the heat release may be different. For example, a rapid release of all the heat stored in the carbide can be achieved, or the rate of this process can be decreased by constantly heating the mixture. It is necessary to maintain a balance between heat release and heat consumption. Therefore, in the ideal case, the process of heat release should be controlled in order to have the possibility to transfer this heat to the reaction mixture but at the same time not too fast (explosive) since, in this case, almost all the heat would be carried away from the reaction mixture with the hot acetylene gas or dissipated.

**Figure 4:**
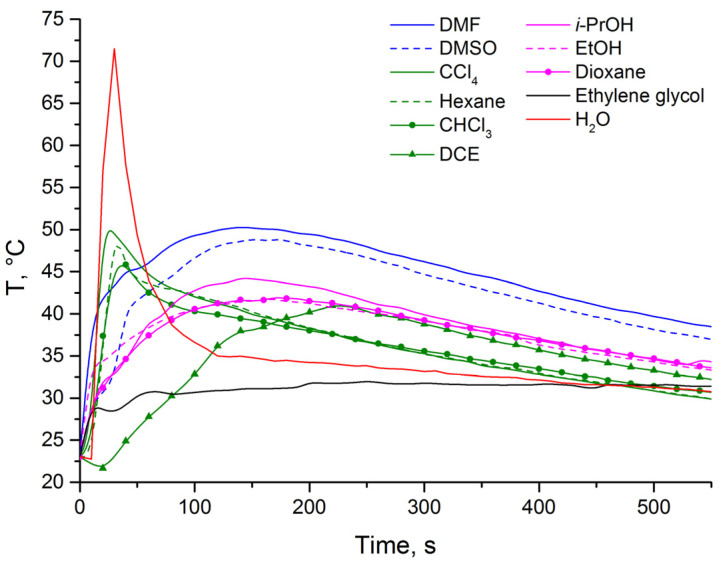
Observed temperature profile for different solvents used in the reaction; conditions: 4 mL of the solvent, 2 mL of water, and 0.56 g of CaC_2_.

Variations in the reaction conditions for different solvent/water mixtures showed that the maximum heating could be achieved in the presence of DMSO and DMF (Figure 4). In the case of the addition of pure water, an explosive release of heat with the dissipation of heat from the reaction mixture was observed, but not the transfer of heat to the desired reactants of the second reactions.

Other water-miscible solvents decreased the maximum temperature (alcohols). Solvents with a higher density, immiscible with water, reduced the rate of heat generation with water without the formation of a homogeneous phase; that is, the carbide was hydrolyzed with water on the surface when it reached water (halogenated solvents: chloroform, CCl_4_, except DCE). The same result was obtained when solvents with lower density were used (e.g., hexane). In that case, water was immediately mixed with carbide, bypassing the solvent layer. Unexpected results were found using ethylene glycol, which, after mixing with water, significantly reduced the rate of hydrolysis. The unusual profile for dichloroethane was explained by its high density, when the carbide simply could not reach the water layer. Thus, by changing the solvent, the kinetic profile of the reaction can be controlled, increasing or decreasing its thermal effect at the initial moment.

**Figure 5:**
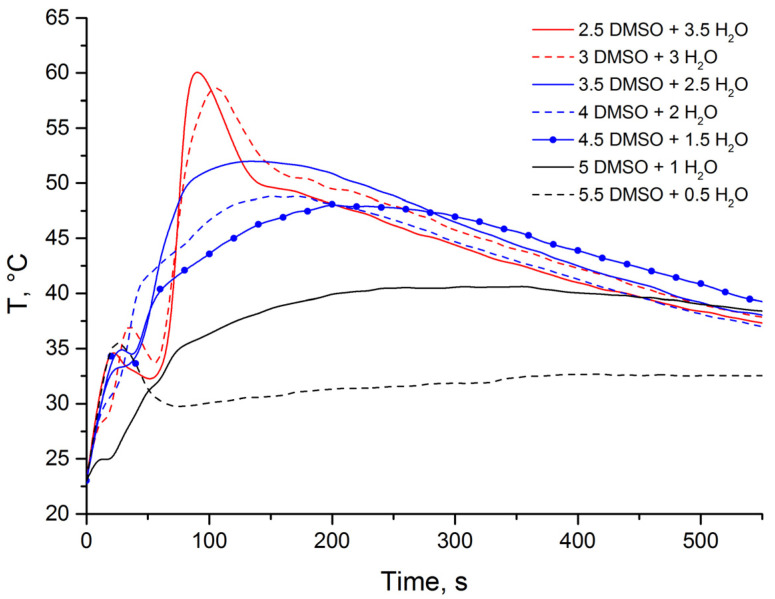
Temperature profile depending on DMSO–water ratios (total volume in all the cases is 6 mL).

For the particular studied reaction, DMSO was chosen as a model solvent because the heat release was significant in DMSO. In addition, many reactions with calcium carbide occur in DMSO, and the usage of DMSO prevents contamination of the reaction mixtures. Varying the DMSO–water ratio led to very interesting results. By changing this ratio, either a rapid release of heat occurs, or this process is significantly reduced and becomes barely noticeable (Figure 5).

This result was expected due to the same amount of carbide and excess water. Thus, the heat profiles of the carbide hydrolysis reaction depend on the DMSO–water ratio. With a small amount of water and a large amount of DMSO, the evolution of heat was practically not observed; that is, it was so slow and prolonged in time that it was accompanied with a minor temperature increase. It should be noted that, in all the cases, there was enough water to hydrolyze all the available carbides (excess water was used in all cases). With an increase in the amount of water in the mixture, the heat release profile changed significantly: the carbide reacted roughly with water with the release of heat in the first few seconds (Figure 5, 3DMSO + 3H_2_O, red dashed curve). Further increasing the proportion of water no longer affected the curve (Figure 5, 2.5DMSO + 3.5H_2_O, red curve). Most likely, water, when added to DMSO, forms sufficiently strong hydrogen bonds with DMSO, which leads to its partial or significant binding. Surprisingly, calcium carbide, with its high dehydrating ability (CaC_2_ is also used as a drying agent), could not consume water from the DMSO–water mixture; more precisely, this process was extremely extended in time. The presence of a semihydrolyzed Ca-containing intermediate is possible, and a carbide molecule is bound to one molecule of water (half-hydrolyzed). This intermediate was solvated by DMSO, which prevents hydrolysis at the second stage [33].

CaC_2_ + nDMSO = [(DMSO)_n_(CaC_2_)] (1)

[(DMSO)_n_(CaC_2_)] + H_2_O = [(DMSO)_n_(Ca(C≡CH)(OH))] (2)

[(DMSO)_n_(Ca(C≡CH)(OH))] + H_2_O = HC≡CH + Ca(OH)_2_ + nDMSO (3)

The solvation of calcium carbide by DMSO was assumed in the reaction mixture (1). When several drops of water are added to the medium, incomplete hydrolysis is observed, and half-hydrolyzed carbide forms a fairly stable intermediate with one water molecule and DMSO as a stabilizer (2). The system is quite stable and can, like a buffer, consume large amounts of water, preventing further hydrolysis of the carbide. With the introduction of a larger excess of water, the formed hemihydrate undergoes full hydrolysis at the second stage. This process occurs quickly and is accompanied by the release of gaseous acetylene and the precipitation of calcium hydroxide (3). In this case, DMSO molecules are released from a coordinated state into a solution. The described hypothesis was confirmed by the hydrolysis of carbide in a 5.5 DMSO/0.5 water mixture (Figure 5, bottom, dashed black line). In this mixture, poor heat evolution was observed because the hydrolysis proceeded very slowly despite the fact that an excess of water was added in relation to the completely hydrolyzed carbide. Nevertheless, when additional amounts of water were added to the system, the hydrolysis completely proceeded with the release of the expected heat.

After optimizing the solvent and the optimal solvent–water ratio conditions, the amount of carbide was varied to achieve the maximum thermal effect. Of course, an increase in carbide loading resulted in an increase in the heat that could be produced. Therefore, the analysis of the heat release from the reaction of one CaC_2_ grain with a pair of drops of a DMSO–water mixture showed an increase in the temperature near the granule surface by 4 to 5 °C. The use of three to five carbide granules leads to an increase in temperature by 10 °C. Note that it is not advisable to load a large excess of carbide since unreacted acetylene will leave the reaction vessel or explode when locked. Therefore, the amount of carbide was varied to assess the best balance between the value of the thermal effect and the duration of heating.

**Figure 6:**
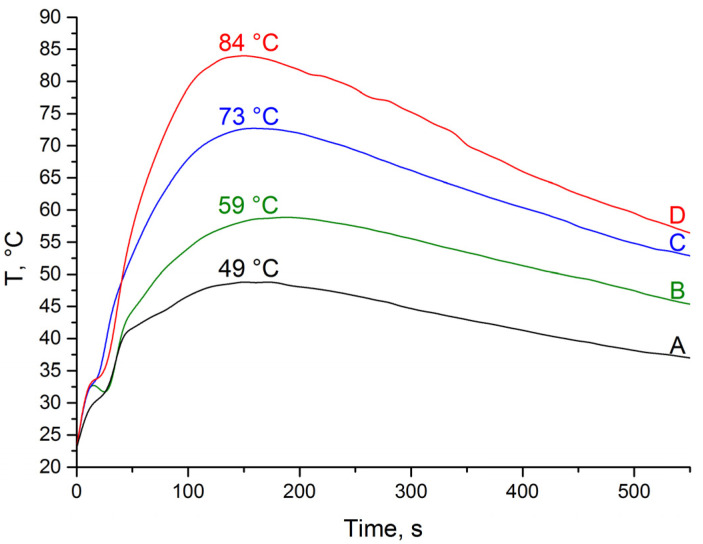
Influence of calcium carbide loading on heat release in the studied mixture (4 mL of DMSO + 2 mL of water). A—carbide loading 0.56 g, B—carbide loading 0.87 g, C—carbide loading 1.28 g, D—carbide loading 1.76 g.

As expected, an increase in carbide loading resulted in an increase in the temperature of the reaction mixture (Figure 6). After the hydrolysis of 1.76 g of carbide, the temperature of the reaction mixture reached about 84 °C (Figure 6, red line). However, heating continued for only 5 min, and the temperature of the reaction mixture decreased rapidly. Attempts to isolate the system and prevent cooling (flask with aluminum foil and cotton) did not significantly change the picture. Nevertheless, a temperature of approximately 80 to 90 °C is easily achievable, and, at this temperature, many processes can be started or those already occurring can be significantly accelerated.

### 2.4. Thiovinylation Reaction. Calcium Carbide as a Double Source of Acetylene and Heat

**Scheme 2:**
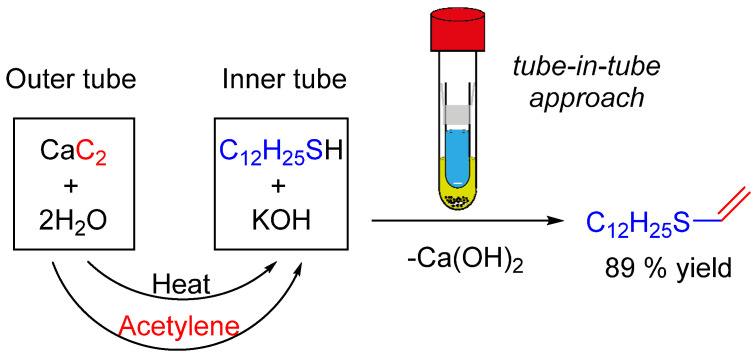
Using calcium carbide as a source of heat and acetylene in the preparation of vinyl sulfide.

The thiovinylation reaction was carried out in two reaction setups: in a two-chamber reactor and in a tube-and-tube reactor (both procedures are described in the Experimental part). The reaction in a two-chamber reactor was necessary to compare the yield of the desired product since, in this case, all the heat from the hydrolysis of calcium carbide was dissipated and was not transferred to the reaction mixture, where thiovinylation occurred. All the loadings and amounts of reagents, solvents, and times were completely the same for both processes. After the completion of the reaction, the mixtures were extracted and studied by NMR with an internal standard. Thus, in a two-chamber reactor, the yield of thiovinyl ether was only 30%, while, in a tube-in-tube reactor, the yield was 89% (Scheme 2). It should be noted that, in both cases, the reactions were not carried out in closed systems. Balloons were attached to the neck of the reactors. On the one hand, it prevented a risk of explosion, and, on the other hand, the extra pressure obtained did not affect the reaction (since reactions with gases are pressure-sensitive). Acetylene is readily soluble in DMSO, especially under high pressure. Therefore, if the reaction would be carried out in a closed system under pressure, then it should be difficult to analyze the effect of heat on the product yield because pressure would also have an influence on this process.

It should be noted that test experiments were carried out by mixing solvents with water in the absence of other reagents and calcium carbide. Additionally, the experiments were carried out using calcium hydroxide instead of calcium carbide to determine that the heat effect of the reaction is due precisely to the hydrolysis of calcium carbide and not to the solvation processes of the obtained calcium hydroxide. These experiments and curves are given in the Supporting Information. The experiments were carried out in several runs. Several temperature profiles were measured, and statistical processing of the results was performed to improve accuracy (see Supplementary Material, Section S5).

## 3. Materials and Methods

### 3.1. General Information

1-Dodecanethiol, ≥98% purity was purchased from Sigma-Aldrich and used without purification. KOH was purchased from local supplier Vecton, Saint Petersburg and was grinded before use. Calcium carbide, product of Germany (granulated, particle size 0.1–1 mm, 75% acetylene (gas volumetric as indicated by the supplier)) was purchased from Sigma-Aldrich and used without further purification. Ca(OH)_2_ was purchased from Vecton, Saint Petersburg, Russia and dried before use. DMSO as a solvent was purchased from Vecton, Saint Petersburg and used as received.

^1^H NMR spectra were recorded using a Bruker Avance 400 NMR spectrometer. Chemical shifts δ are reported in ppm relative to residual CHCl_3_ and CDCl_3_ as internal standards. The data were processed using MestReNova (version 6.0.2) desktop NMR data processing software. Toluene was used as the internal standard in the ^1^H NMR spectra to determine the yields of thiovinyl ether.

A thermal imager CEM DT-870 was used to register the temperature inside a reactor. The temperature curves were automatically created by a thermal imager.

### 3.2. General Procedures

*Optimization experiments*. 1 mL of DMSO was poured into the inner (small) tube, 0.56 g of carbide was loaded into the outer (large) tube, and then DMSO was placed in the outer tube. A liner was inserted into the outer tube, and the inner tube was immersed inside the outer tube and fixed. An appropriate volume of water was injected into the carbide-containing tube from the syringe along the wall. The thermal imager was turned on before adding water, and data were recorded every 10 s for 10 min.

In experiments with calcium carbide, each experiment was repeated at least 3 times, and the resulting curves were added to each other to reduce the difference in the delay at the start of the experiments. The reproducibility of the experiments was checked using the Korchen test (the significance level was 0.05. See Supplementary Material, Section S5). In the case of exceeding the obtained value of the criterion compared with the standard, additional experiments were carried out at the corresponding points.

Runs with calcium hydroxide were carried out according to the procedure described for carbide, replacing carbide with calcium hydroxide (0.49 g). The ratio of solvent:water was 4:2, and the total volume was always 6 mL (so that liquid levels in the inner and outer tubes were the same. See Supplementary Material, Section S2.

Blank experiments were performed by mixing a solvent with water using a tube-in-tube reactor. The experiments were carried out only for solvents that showed nonzero heat generation in experiments with calcium hydroxide. The experiment with calcium hydroxide and the blank was carried out once.

General procedure for the vinylation of dodecanthiol in a tube-in-tube reactor: 100 μL (0.42 mmol) of dodecanethiol, 0.8 mL of DMSO, and 25 mg (0.45 mmol) of KOH were placed into an inner tube with a small stirrer. Calcium carbide (1.76 g) was placed into an outer tube with a stirrer, and 4 mL of DMSO was added. The liner was inserted, and 2 mL of water was slowly added along the wall using a syringe. The tube-in-tube reactor was closed with a balloon and placed in insulation made of cotton wool and foil equipped with a thermocouple. The reaction mixture was stirred at 500 rpm for 3 h. Then, the inner tube was removed, and the reaction mixture was diluted with 5% sodium hydroxide solution and extracted 3 times with an equal amount of diethyl ether. The organic layer was washed 3 times with brine and dried over sodium sulfate, and then the solvent was evaporated. The yield of vinyl ether was determined by ^1^H NMR using an internal standard (toluene); see Supplementary Material, Section S6).

General procedure for the vinylation of dodecanthiol in a two-chamber reactor: the runs were performed in a two-chamber reactor in the same manner using the same loadings with one exception: carbide, water, and DMSO were loaded in one chamber, and the initial thiol, DMSO and a base were loaded in another.

## 4. Conclusions

In this work, we have demonstrated that calcium carbide can be successfully used not only as a source of gaseous acetylene but also as a heat source. The heat released during hydrolysis can be consumed for the other chemical reaction, which leads to an increase in the yield of the desired product from 30% to 89%. Thus, the carbide is able to promote and accelerate reactions without external heat sources. The kinetics of the hydrolysis of calcium carbide by mixtures of various solvents with water are different: it is possible to achieve rapid spontaneous heat release, slow, and gradual. Quenching a carbide with water may be less effective than adding a mixture of solvents. When quenched with water, a high temperature is immediately reached, but a significant part of the heat is lost. In addition, acetylene polymerizes at high temperatures, the polymerization products contaminate the target product, and the system has to be purified. Hydrolysis with a mixture of solvents makes the process controllable and the heat transfer process more efficient. Varying a cosolvent, the rate of carbide hydrolysis can be easily controlled. The proposed tube-in-tube reactor ensures that reactions are carried out in a more efficient manner since the heat generated is also used to promote the reaction.

To summarize, the key results may be described as follows: (i) temperature profiles of the selected reactions with calcium carbide were measured; (ii) dependences of the thermal effects of carbide hydrolysis on cosolvents were revealed; (iii) the dependence of the thermal effect of carbide hydrolysis on the water/solvent ratio was also revealed; (iv) effectiveness of calcium carbide as a simultaneous source of heat and acetylene was demonstrated in the thiovinylation reaction.

Here, we describe a general idea of energy-economic transformation using the example of calcium carbide. Many other energy-rich reagents, such as NaBH_4_, DIBAL, LAH, Grignard reagents, etc., are used in modern chemical transformations. Not limited to the reagents, a negative standard enthalpy change is characteristic of several reactions, such as combining acids and bases, addition reactions, and oxidation. Therefore, the methodology described here may be useful for a number of different reactions. The developed design of the liners can be used not only to study the temperature profiles of reactions. The tube-in-tube methodology opens a number of new opportunities in practical applications as it is a cost-effective development for H-tube and/or two-chamber reactors [34,35,36,37,38,39,40,41,42,43,44,45,46]. The simple design, inexpensive components, and easy-to-handle loading/retrieval of chemicals are valuable advantages in complicated reactions with a shared gaseous atmosphere [47].

## Data Availability

Data are contained in the article and Supplementary Materials.

## Supplementary Materials

The following supporting information can be downloaded at: https://www.mdpi.com/article/10.3390/ijms23052763/s1.
