# Supplementary material for: Thermal Mapping of Self-Promoted Calcium Carbide Reactions for Performing Energy-Economic Processes
Source: Int J Mol Sci. 2022 Mar 2;23(5):2763. doi: 10.3390/ijms23052763 (PMC8911359; doi:10.3390/ijms23052763)
Supplement: Supplementary file 1 [file ijms-23-02763-s001.zip › ijms-1584214-supplementary.pdf]

Supporting Information

for the article

Thermal Mapping of Self-Promoted Calcium Carbide Reactions for Performing Energy-Economic Processes

Konstantin S. Rodygin,[a] Kristina A. Lotsman,[a] Kirill S. Erokhin,[b] Viktoria A. Korabelnikova,[b] Valentine P. Ananikov*[a,b]

[a] Institute of Chemistry, Saint Petersburg State University, Universitetskiy pr. 26, Stary Petergof 198504, Russia.

[b] Zelinsky Institute of Organic Chemistry, Russian Academy of Sciences, Leninsky pr. 47, Moscow 119991, Russia. E-mail: val@ioc.ac.ru

Contents

1. Design of the liner....S3
2. Blank experiments with calcium hydroxide S5
3. Blank experiments in the absence of calcium carbide S6
4. Blank experiments with DMSO/water mixture in the absence of reagents....S7
5. Statistical processing of the results S8
6. NMR data S44
7. Reaction setup....S45
8. Monitoring of the heating with a thermocouple S46

1. Design of the liner

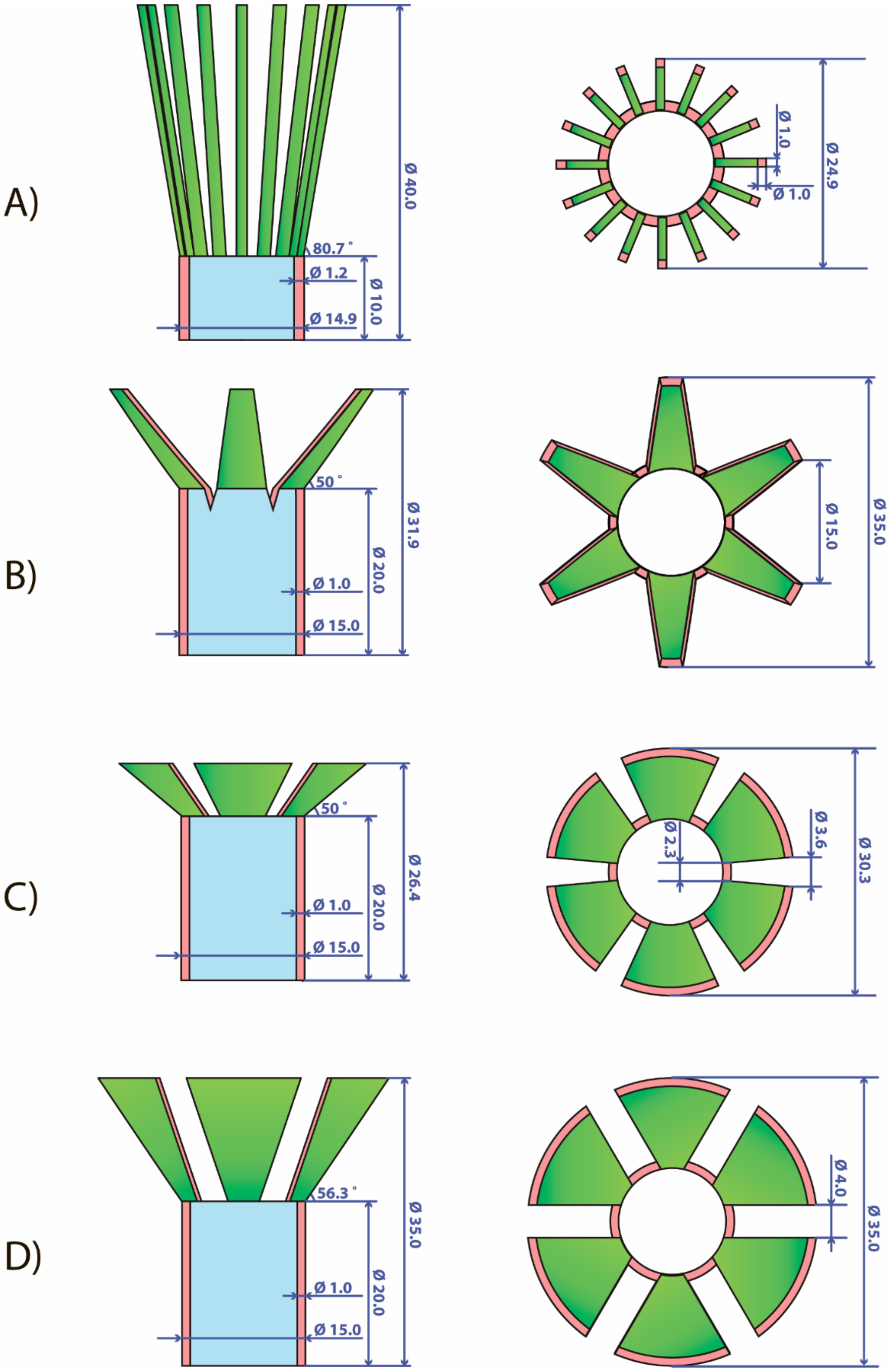

Figure S1: Variety of analysed liners.

The liner consists of two main parts: an inner tube retainer and a position retainer. The inner tube retainer is a hollow cylinder, the inner diameter of which is determined by the outer diameter of the inner tube. Position retainer is a set of “petals”. The strength of tube-in-tube fixation depends on the contact area and pressure force of the petals,

which are determined by the size of the petals and the angle between them and the hollow cylinder. The performance of a variety of 3D printed liners was analyzed. In the case of small petals and a high angle (Fig. S1A), the liner was unable to fix the position of the tube, so the tube fell immediately. Decreasing the angle slightly improved the performance (Fig. S1B, C), resulting in short-term fixation. Although, the inner tube fell too. Increasing the length of the petals with a simultaneous increase in the outer diameter at the end point and contact area led to the creation of a liner that allowed the tube to be fixed throughout the experiment (Fig. S1D).

3D-printing options. The liner was printed using a Solo Master 3D printer equipped with a 0.4 mm nozzle. A commercially available nylon filament (Best Filament trade mark) with a 1.75 mm diameter was used. The options were as follows:

- Layer height: 0.1 mm
- Extrusion width: 0.4 mm
- Temperature of nozzle: 230 °C
- Extrusion multiplier: 1.00
- Z-offset: 0.00

2. Blank experiments with calcium hydroxide

Calcium hydroxide formed after calcium carbide hydrolysis. The solvation of the hydroxide by a solvent is possible. To examine the effect of calcium hydroxide solvation, the temperature profile of $Ca(OH)_2$ with various solvents was recorded. The profiles of the mixing were the same as in the case of blank experiments with water in the absence of any additives. Thus, the increasing of temperature was caused by mixing water with a solvent but not by solvation of calcium hydroxide.

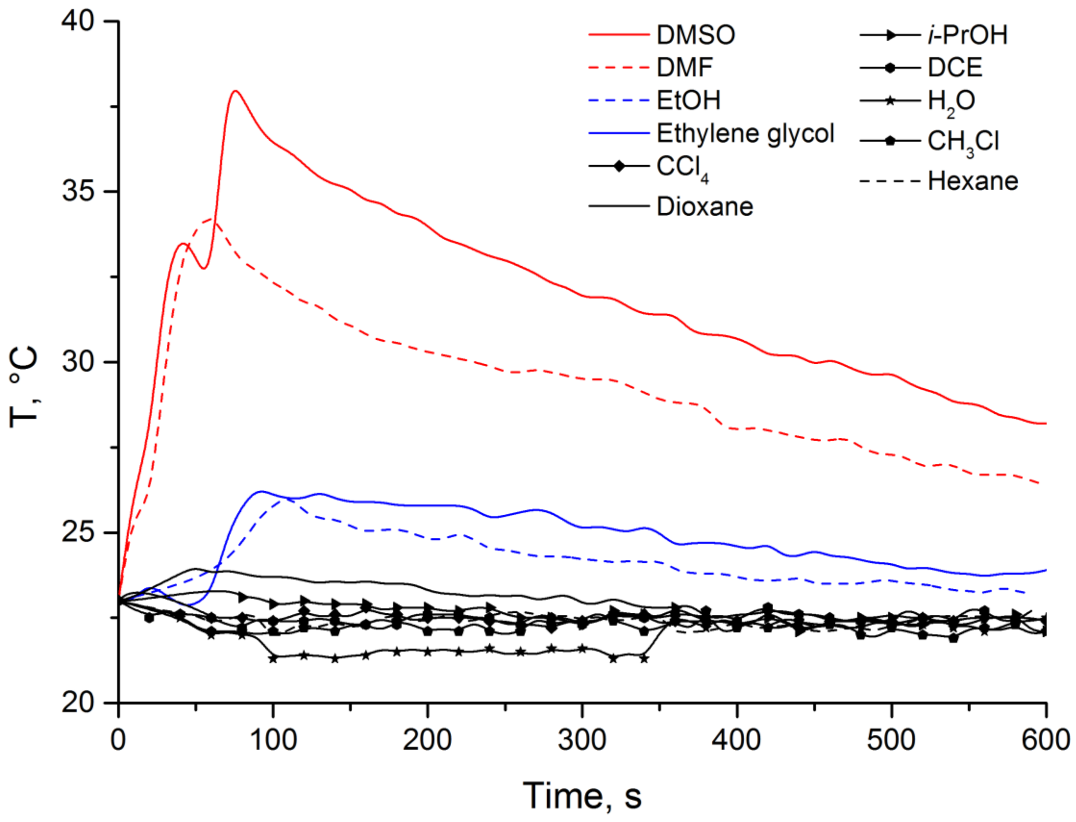

Figure S2: Heat generation in a tube-in-tube reactor with calcium hydroxide (the observed temperature change was due to mixing DMSO and water, see Sections 3, 4).

3. Blank experiments in the absence of calcium carbide

Solvents may form hydrogen bonds with water when mixed. Blank experiments were carried out to measure heating in the absence of calcium carbide and calcium hydroxide. As a result, the increasing of temperature was cause by mixing water and a solvent.

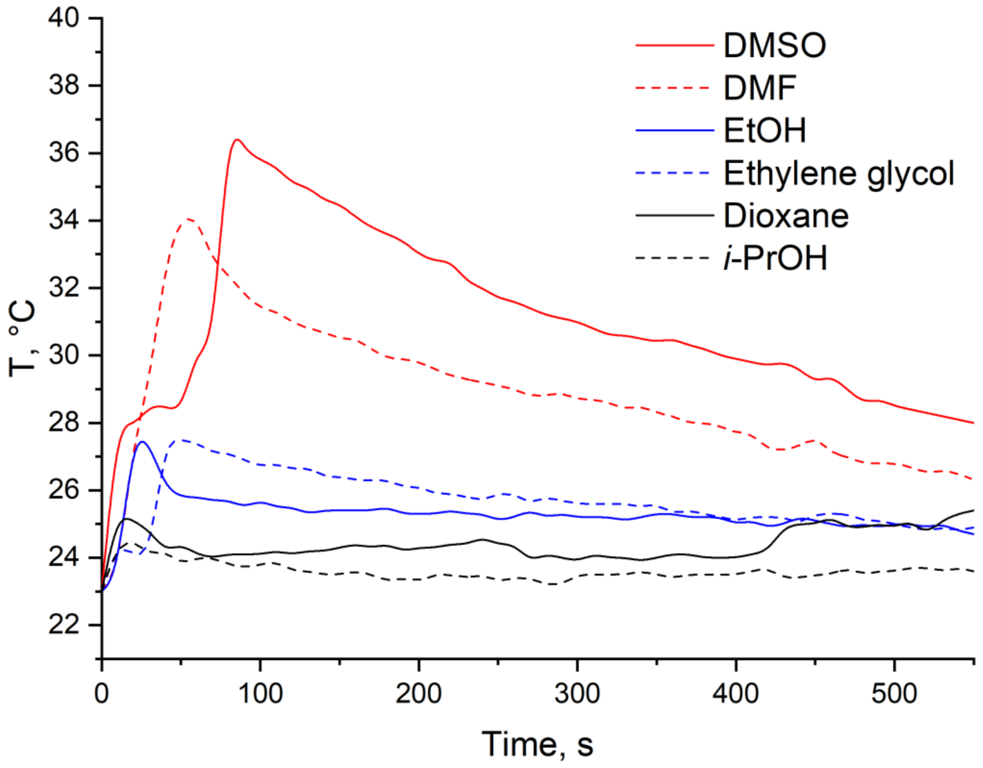

Figure S3: Heat generation in a tube-in-tube reactor in the absence of calcium carbide and calcium hydroxide.

4. Blank experiments with DMSO/water mixture in the absence of reagents

A reaction tube was charged with DMSO (standard amount). A thermometer was placed right into the solvent. The starting temperature was 24 °C. Then, an appropriate amount of water was dropped into the solvent, and stirring was initiated (500 rpm). The temperature increased to 43 °C (the maximum) and started to decrease. After 10 minutes, the temperature was 34 °C.

5. Statistical processing of the results

To ensure in the accuracy of the measurements statistical processing was carried out.

D(T) - S_m^2

S – standard deviation of data series *m*

G = max D(T)/Sum D(T)

The temperature profile of the reaction was depended on the starting time of stirring after the addition of water. Therefore, the starting time of stirring was varied from 0 seconds (right after the addition water the stirring was turned on) to 60 seconds.

Then, the other conditions were varied (type of solvent, loadings, water/solvent ratio).

All the experiments were carried out using a standard procedure (please, see section 3.2. General Procedures in the Manuscript).

Table S1: Time variation, 0 s.

0 s					
Time, s	T_1, °C	T_2, °C	T_3, °C	T_{med}, °C	D(T)
0	23.0	23.0	23.0	23.0	0.00
10	41.4	39.4	42.3	41.0	2.20
20	42.0	43.5	43.3	42.9	0.66
30	42.5	43.5	43.9	43.3	0.52
40	43.8	44.4	44.9	44.4	0.30
50	44.7	45.2	45.7	45.2	0.25
60	45.9	46.1	46.3	46.1	0.04
70	46.6	47.1	46.8	46.8	0.06
80	47.5	47.8	47.2	47.5	0.09
90	47.9	48.5	47.6	48.0	0.21
100	48.5	48.6	47.7	48.3	0.24
110	48.8	49.0	48.0	48.6	0.28
120	48.7	49.1	48.1	48.6	0.25
130	49.0	49.4	48.5	49.0	0.20
140	49.2	49.4	48.8	49.1	0.09
150	49.1	49.2	48.6	49.0	0.10
160	49.0	49.3	48.7	49.0	0.09
170	48.4	49.1	48.3	48.6	0.19
180	48.7	48.8	48.6	48.7	0.01
190	48.2	48.7	47.9	48.3	0.16
200	48.2	48.4	47.9	48.2	0.06
210	48.1	48.5	47.7	48.1	0.16
220	47.5	47.9	47.4	47.6	0.07
230	47.1	47.7	47.1	47.3	0.12
240	46.8	47.3	46.9	47.0	0.07
250	46.7	46.5	46.7	46.6	0.01

260	46.3	46.2	46.6	46.4	0.04
270	45.9	45.8	46.4	46.0	0.10
280	45.3	45.4	46.3	45.7	0.30
290	45.2	44.9	46.1	45.4	0.39
300	44.5	44.8	45.4	44.9	0.21
310	44.1	44.5	45.9	44.8	0.89
320	43.6	43.9	44.9	44.1	0.46
330	43.1	43.5	44.6	43.7	0.60
340	43.0	43.3	44.4	43.6	0.54
350	42.3	42.8	44.0	43.0	0.76
360	42.1	42.5	43.7	42.8	0.69
370	41.6	42.0	43.3	42.3	0.79
380	41.5	41.8	43.0	42.1	0.63
390	41.1	41.5	42.9	41.8	0.89
400	40.7	41.1	42.4	41.4	0.79
410	40.4	40.7	42.3	41.1	1.04
420	40.0	40.4	41.9	40.8	1.00
430	39.6	40.0	41.7	40.4	1.24
440	39.3	39.8	41.4	40.2	1.20
450	38.9	39.7	41.1	39.9	1.24
460	38.6	39.3	40.7	39.5	1.14
470	38.4	38.9	40.4	39.2	1.08
480	38.2	38.6	40.0	38.9	0.89
490	37.9	38.4	39.7	38.7	0.86
500	37.6	38.0	39.3	38.3	0.79
510	37.3	37.8	39.4	38.2	1.20
520	37.2	37.7	39.2	38.0	1.08
530	36.8	37.3	38.9	37.7	1.20
540	36.4	37.0	38.5	37.3	1.17
550	36.2	36.9	38.3	37.1	1.14
Sample variance					0.55
				max D(T) =	2.20
				min D(T) =	0
				G=	0.0713
				Sum D(T)=	30.89
				G known=	0.1131

Table S2: Time variation, 10 s.

10 s					
Time, s	T_1, °C	T_2, °C	T_3, °C	T_{med}, °C	D(T)
0	23.0	23.0	23.0	23.0	0.00
10	31.7	32.6	30.2	31.5	1.47
20	39.4	39.3	39.6	39.4	0.02
30	39.5	40.5	41.0	40.3	0.58
40	40.7	41.5	42.0	41.4	0.43

50	42.2	42.5	43.2	42.6	0.26
60	43.0	43.4	44.4	43.6	0.52
70	44.2	44.2	45.0	44.5	0.21
80	45.2	45.3	45.7	45.4	0.07
90	45.7	45.7	46.2	45.9	0.08
100	46.3	46.1	46.8	46.4	0.13
110	46.9	46.7	47.4	47.0	0.13
120	47.1	47.0	47.5	47.2	0.07
130	47.3	46.9	47.4	47.2	0.07
140	47.1	47.1	47.1	47.1	0.00
150	47.0	47.2	47.1	47.1	0.01
160	46.6	47.2	46.9	46.9	0.09
170	46.5	46.9	46.5	46.6	0.05
180	46.1	46.5	46.6	46.4	0.07
190	46.1	46.2	46.1	46.1	0.00
200	45.7	45.9	45.4	45.7	0.06
210	45.5	45.7	45.3	45.5	0.04
220	45.2	45.5	44.8	45.2	0.12
230	44.7	45.2	44.7	44.9	0.08
240	44.4	44.9	44.3	44.5	0.10
250	44.1	44.3	44.0	44.1	0.02
260	43.8	44.0	43.5	43.8	0.06
270	43.4	43.5	43.1	43.3	0.04
280	43.1	43.0	42.7	42.9	0.04
290	42.6	43.0	42.3	42.6	0.12
300	42.2	42.2	42.1	42.2	0.00
310	42.0	42.2	42.0	42.1	0.01
320	41.4	41.7	41.5	41.5	0.02
330	40.8	41.5	40.6	41.0	0.22
340	40.8	41.2	40.2	40.7	0.25
350	40.4	40.9	39.8	40.4	0.30
360	40.8	40.6	39.5	40.3	0.49
370	40.4	40.2	39.0	39.9	0.57
380	40.1	39.8	38.8	39.6	0.46
390	39.8	39.5	38.5	39.3	0.46
400	39.4	39.6	38.3	39.1	0.49
410	39.2	39.3	38.1	38.9	0.44
420	39.0	38.9	37.9	38.6	0.37
430	38.7	38.3	37.8	38.3	0.20
440	38.4	38.1	37.4	38.0	0.26
450	38.3	37.9	36.9	37.7	0.52
460	37.9	37.5	36.4	37.3	0.60
470	37.4	37.3	36.3	37.0	0.37
480	37.4	37.0	36.0	36.8	0.52
490	36.6	36.9	35.7	36.4	0.39

500	35.7	36.7	35.4	35.9	0.46
510	35.6	36.2	35.3	35.7	0.21
520	35.1	36.1	35.0	35.4	0.37
530	35.4	35.7	34.9	35.3	0.16
540	35.0	35.8	34.7	35.2	0.32
550	34.7	35.2	34.3	34.7	0.20
560	34.5	34.9	34.1	34.5	0.16
570	34.3	34.8	34.0	34.4	0.16
580	34.3	34.7	33.4	34.1	0.44
Sample variance					0.25
				max D(T) =	1.47
				min D(T) =	23
				G=	0.1015
				C Sum D(T)=	14.48
				G known=	0.1131

Table S3: Time variation, 20 s.

20 s					
Time, s	T_1, °C	T_2, °C	T_3, °C	T_{med}, °C	D(T)
0	23.0	23.0	23.0	23.0	0.00
10	30.1	29.8	28.2	29.4	1.04
20	32.0	30.1	29.8	30.6	1.42
30	32.1	32.8	31.8	32.2	0.26
40	40.0	40.5	41.5	40.7	0.58
50	41.1	41.1	42.5	41.6	0.65
60	42.6	42.7	42.7	42.7	0.00
70	44.1	43.0	43.9	43.7	0.34
80	45.2	43.0	44.9	44.4	1.42
90	46.2	44.9	45.9	45.7	0.46
100	46.9	46.4	46.7	46.7	0.06
110	47.7	47.4	47.3	47.5	0.04
120	48.2	48.2	47.9	48.1	0.03
130	48.7	48.6	47.9	48.4	0.19
140	48.8	48.8	48.1	48.6	0.16
150	49.1	49.1	48.5	48.9	0.12
160	48.8	48.6	48.6	48.7	0.01
170	49.0	48.7	48.8	48.8	0.02
180	48.7	48.6	48.9	48.7	0.02
190	48.5	48.2	48.1	48.3	0.04
200	48.0	48.1	48.1	48.1	0.00
210	48.0	47.8	47.8	47.9	0.01
220	47.7	47.3	47.9	47.6	0.09
230	47.5	47.2	47.3	47.3	0.02
240	47.3	46.9	46.9	47.0	0.05
250	46.9	46.2	46.9	46.7	0.16

260	46.5	45.9	46.6	46.3	0.14
270	45.8	45.7	46.2	45.9	0.07
280	45.8	45.3	45.9	45.7	0.10
290	44.8	44.9	45.4	45.0	0.10
300	44.4	44.5	45.2	44.7	0.19
310	43.9	44.3	44.6	44.3	0.12
320	43.6	44.0	44.5	44.0	0.20
330	43.3	43.5	44.0	43.6	0.13
340	42.8	43.3	43.8	43.3	0.25
350	42.6	42.7	43.5	42.9	0.24
360	42.2	42.5	43.0	42.6	0.16
370	41.8	42.5	42.7	42.3	0.22
380	41.5	42.0	42.4	42.0	0.20
390	41.5	41.3	42.0	41.6	0.13
400	41.1	41.2	41.6	41.3	0.07
410	40.5	40.9	41.3	40.9	0.16
420	40.1	40.6	41.0	40.6	0.20
430	39.9	40.1	40.7	40.2	0.17
440	39.6	39.8	40.4	39.9	0.17
450	39.1	39.7	40.0	39.6	0.21
460	39.2	39.3	39.8	39.4	0.10
470	38.5	39.0	39.4	39.0	0.20
480	38.2	38.8	39.1	38.7	0.21
490	38.1	38.3	38.9	38.4	0.17
500	37.7	38.0	38.7	38.1	0.26
510	37.5	37.7	38.3	37.8	0.17
520	37.4	37.5	38.1	37.7	0.14
530	37.2	37.4	37.9	37.5	0.13
540	36.8	37.0	37.9	37.2	0.34
550	36.5	36.7	37.7	37.0	0.41
560	36.3	36.6	37.4	36.8	0.32
570	36.1	36.3	37.4	36.6	0.49
580	35.9	36.0	37.7	36.5	1.02
590	35.8	35.6	37.4	36.3	0.97
Sample variance					0.26
				max D(T) =	1.42
				min D(T) =	0
				G=	0.0918
				Sum D(T)=	15.50
				G known=	0.1131

Table S4: Time variation, 60 s.

60 s					
Time, s	T_1, °C	T_2, °C	T_3, °C	T_{med}, °C	D(T)
0	23.0	23.0	23.0	23.0	0.00

10	28.9	27.6	28.2	28.2	0.42
20	28.6	27.2	28.1	28.0	0.50
30	28.4	26.8	27.2	27.5	0.69
40	28.1	26.5	27.4	27.3	0.64
50	27.9	26.7	26.9	27.2	0.41
60	27.8	28.5	29.7	28.7	0.92
70	36.0	37.6	38.8	37.5	1.97
80	38.9	39.9	39.6	39.5	0.26
90	39.2	39.6	39.8	39.5	0.09
100	40.7	39.5	40.4	40.2	0.39
110	41.4	40.6	41.4	41.1	0.21
120	42.4	41.3	42.3	42.0	0.37
130	43.5	42.0	43.0	42.8	0.58
140	44.1	43.0	43.5	43.5	0.30
150	44.7	43.5	44.4	44.2	0.39
160	45.3	44.3	44.6	44.7	0.26
170	45.4	44.7	44.8	45.0	0.14
180	45.3	45.1	45.1	45.2	0.01
190	45.6	45.4	45.2	45.4	0.04
200	45.7	45.9	45.4	45.7	0.06
210	45.8	46.2	45.5	45.8	0.12
220	45.7	45.7	45.4	45.6	0.03
230	45.6	45.9	45.1	45.5	0.16
240	45.2	46.3	45.1	45.5	0.44
250	45.1	46.2	44.7	45.3	0.60
260	44.7	46.0	44.6	45.1	0.61
270	44.7	45.9	44.4	45.0	0.63
280	44.4	46.0	44.5	45.0	0.80
290	43.9	45.8	44.2	44.6	1.04
300	43.7	45.6	43.9	44.4	1.09
310	43.4	45.5	43.7	44.2	1.29
320	43.2	45.2	43.5	44.0	1.16
330	43.1	45.0	43.1	43.7	1.20
340	42.7	44.7	42.8	43.4	1.27
350	41.7	44.9	42.6	43.1	2.72
360	41.5	44.5	42.2	42.7	2.46
370	41.4	44.2	42.0	42.5	2.17
380	40.9	43.9	41.7	42.2	2.41
390	40.5	43.7	41.1	41.8	2.89
400	40.2	43.5	40.8	41.5	3.09
410	39.8	43.1	40.5	41.1	3.02
420	39.6	42.9	40.1	40.9	3.16
430	39.2	42.5	40.0	40.6	2.96
440	39.1	42.1	39.5	40.2	2.65
450	38.7	41.8	39.2	39.9	2.77

460	38.5	41.6	39.0	39.7	2.77
470	38.3	41.1	38.8	39.4	2.23
480	38.0	41.1	38.5	39.2	2.77
490	37.6	41.1	38.3	39.0	3.43
500	37.4	40.7	38.0	38.7	3.09
510	37.0	40.5	37.3	38.3	3.76
520	36.7	40.4	37.0	38.0	4.22
530	36.3	39.3	36.8	37.5	2.58
540	36.4	39.2	36.5	37.4	2.52
550	36.0	38.7	36.4	37.0	2.12
560	35.7	38.7	36.0	36.8	2.73
570	35.5	38.2	35.9	36.5	2.12
Sample variance					1.45
				max D(T) =	4.22
				min D(T) =	0
				G=	0.0504
				Sum D(T)=	83.86
				G known=	0.1131

Table S5: Solvent variation, chloroform.

$CHCl_3$					
Time, s	T_1, °C	T_2, °C	T_3, °C	T_{med}, °C	D(T)
0	23.0	23.0	23.0	23.0	0.00
10	25.6	25.5	22.4	24.5	3.31
20	37.9	37.3	36.9	37.4	0.25
30	46.3	46.8	44.5	45.9	1.46
40	46.9	45.2	45.4	45.8	0.86
50	44.6	43.4	43.6	43.9	0.41
60	43.5	41.9	42.1	42.5	0.76
70	42.5	41.1	41.5	41.7	0.52
80	41.8	40.6	40.9	41.1	0.39
90	41.1	40.3	40.3	40.6	0.21
100	40.4	40.2	40.3	40.3	0.01
110	40.0	40.1	40.1	40.1	0.00
120	39.6	39.9	40.3	39.9	0.12
130	39.4	39.8	39.8	39.7	0.05
140	39.0	39.5	39.8	39.4	0.16
150	39.0	39.3	39.5	39.3	0.06
160	38.8	39.2	39.2	39.1	0.05
170	38.3	38.9	38.9	38.7	0.12
180	37.8	38.6	39.0	38.5	0.37
190	37.5	38.6	38.5	38.2	0.37
200	37.4	38.4	38.3	38.0	0.30
210	36.9	38.3	38.1	37.8	0.57
220	36.9	38.0	37.9	37.6	0.37

230	36.4	37.8	37.6	37.3	0.57
240	36.2	37.5	37.3	37.0	0.49
250	35.9	37.3	36.9	36.7	0.52
260	35.7	37.2	36.5	36.5	0.56
270	35.4	36.9	36.5	36.3	0.60
280	35.1	36.7	36.4	36.1	0.72
290	34.8	36.5	36.0	35.8	0.76
300	34.7	36.2	35.8	35.6	0.60
310	34.5	36.0	35.7	35.4	0.63
320	34.5	35.9	35.2	35.2	0.49
330	34.1	35.8	35.0	35.0	0.72
340	33.7	35.5	34.7	34.6	0.81
350	33.5	35.4	34.6	34.5	0.91
360	33.3	35.1	34.4	34.3	0.82
370	33.1	35.0	34.1	34.1	0.90
380	32.9	34.8	33.9	33.9	0.90
390	32.6	34.8	33.7	33.7	1.21
400	32.4	34.5	33.5	33.5	1.10
410	32.3	34.3	33.3	33.3	1.00
420	32.0	34.1	33.0	33.0	1.10
430	31.7	33.8	32.8	32.8	1.10
440	31.6	33.6	32.5	32.6	1.00
450	31.5	33.6	32.5	32.5	1.10
460	31.2	32.8	32.3	32.1	0.67
470	31.2	32.7	32.0	32.0	0.56
480	31.0	32.5	31.7	31.7	0.56
490	30.8	32.3	31.7	31.6	0.57
500	30.6	32.2	31.5	31.4	0.64
510	30.4	32.1	31.3	31.3	0.72
520	30.1	31.9	31.2	31.1	0.82
530	30.0	31.8	31.0	30.9	0.81
540	30.0	31.6	31.0	30.9	0.65
550	29.9	31.4	30.7	30.7	0.56
560	29.9	31.5	30.5	30.6	0.65
570	29.4	31.4	30.5	30.4	1.00
Sample variance					0.66
				max D(T) =	3.31
				min D(T) =	0
				G=	0.0879
				Sum D(T)=	37.67
				G known=	0.1131

Table S6: Solvent variation, DMF.

DMF					
Time, s	T_1, °C	T_2, °C	T_3, °C	T_{med}, °C	D(T)

0	23.0	23.0	23.0	23.0	0.00
10	38.8	39.4	38.5	38.9	0.21
20	41.7	43.4	41.2	42.1	1.33
30	42.7	44.2	42.7	43.2	0.75
40	45.0	44.7	45.8	45.2	0.32
50	46.9	43.1	45.7	45.2	3.77
60	48.0	45.1	44.8	46.0	3.12
70	49.0	46.4	46.3	47.2	2.34
80	49.6	47.3	47.7	48.2	1.51
90	49.8	48.1	48.7	48.9	0.74
100	50.3	48.5	49.2	49.3	0.82
110	50.1	48.9	49.7	49.6	0.37
120	50.3	49.4	50.2	50.0	0.24
130	50.5	49.8	50.1	50.1	0.12
140	50.4	50.0	50.5	50.3	0.07
150	50.2	49.9	50.5	50.2	0.09
160	50.0	50.0	50.2	50.1	0.01
170	49.9	49.9	50.4	50.1	0.08
180	49.5	49.8	50.3	49.9	0.16
190	49.0	49.5	50.1	49.5	0.30
200	48.9	49.7	49.8	49.5	0.24
210	48.7	49.4	49.8	49.3	0.31
220	48.2	49.3	49.2	48.9	0.37
230	47.9	49.0	49.3	48.7	0.54
240	47.5	48.7	48.8	48.3	0.52
250	47.2	48.4	48.4	48.0	0.48
260	46.5	48.1	47.9	47.5	0.76
270	46.2	47.7	47.4	47.1	0.63
280	45.9	47.7	47.1	46.9	0.84
290	45.6	47.1	46.7	46.5	0.60
300	45.1	46.9	46.6	46.2	0.93
310	44.8	46.5	46.2	45.8	0.82
320	44.5	46.3	45.9	45.6	0.89
330	44.1	45.4	45.7	45.1	0.72
340	43.7	45.2	45.3	44.7	0.80
350	44.0	44.7	44.8	44.5	0.19
360	43.3	44.7	44.8	44.3	0.70
370	43.1	44.2	44.3	43.9	0.44
380	42.7	43.8	44.1	43.5	0.54
390	42.4	43.3	43.5	43.1	0.34
400	41.9	42.9	43.2	42.7	0.46
410	41.8	42.6	42.8	42.4	0.28
420	41.4	42.3	42.2	42.0	0.24
430	41.1	42.0	42.0	41.7	0.27
440	40.5	41.8	41.8	41.4	0.56

450	40.0	41.5	41.6	41.0	0.80
460	39.7	41.2	41.2	40.7	0.75
470	39.5	40.9	40.8	40.4	0.61
480	39.3	40.8	40.7	40.3	0.70
490	39.1	40.5	40.4	40.0	0.61
500	38.9	40.0	40.1	39.7	0.44
510	38.7	39.8	39.9	39.5	0.44
520	38.2	39.6	39.7	39.2	0.70
530	38.0	39.2	39.4	38.9	0.57
540	37.9	39.1	38.9	38.6	0.41
550	37.6	38.9	39.1	38.5	0.66
560	37.4	38.4	38.7	38.2	0.46
570	37.4	38.3	38.7	38.1	0.44
Sample variance					0.66
				max D(T) =	3.77
				min D(T) =	0
				G=	0.1005
				Sum D(T)=	37.54
				G known=	0.1131

Table S7: Solvent variation, CCl_4.

CCl_4					
Time, s	T_1, °C	T_2, °C	T_3, °C	T_{med}, °C	D(T)
0	23.0	23.0	23.0	23.0	0.00
10	25.2	27.0	26.5	26.2	0.86
20	49.8	52.1	51.3	51.1	1.36
30	48.5	49.4	50.6	49.5	1.11
40	46.8	47.7	49.3	47.9	1.60
50	45.8	45.5	46.9	46.1	0.54
60	44.9	43.9	45.0	44.6	0.37
70	44.2	43.2	43.7	43.7	0.25
80	43.4	42.7	42.9	43.0	0.13
90	43.0	42.4	42.3	42.6	0.14
100	42.5	41.9	41.6	42.0	0.21
110	42.1	41.6	41.2	41.6	0.20
120	41.6	41.4	40.6	41.2	0.28
130	41.3	41.1	40.3	40.9	0.28
140	40.7	40.8	39.9	40.5	0.24
150	40.0	40.2	39.5	39.9	0.13
160	39.8	40.1	39.0	39.6	0.32
170	39.4	39.7	38.8	39.3	0.21
180	39.1	39.4	38.4	39.0	0.26
190	38.7	39.1	37.9	38.6	0.37
200	38.3	38.8	37.7	38.3	0.30
210	38.0	38.5	37.4	38.0	0.30

220	37.8	38.0	37.2	37.7	0.17
230	37.5	37.5	36.6	37.2	0.27
240	37.1	37.0	36.4	36.8	0.14
250	36.9	36.9	36.2	36.7	0.16
260	36.7	36.4	35.9	36.3	0.16
270	36.3	36.2	35.6	36.0	0.14
280	36.1	36.0	35.1	35.7	0.30
290	35.7	35.8	35.0	35.5	0.19
300	35.4	35.7	34.8	35.3	0.21
310	35.1	35.4	34.4	35.0	0.26
320	34.8	35.0	34.1	34.6	0.22
330	34.5	35.0	34.0	34.5	0.25
340	34.4	34.7	33.8	34.3	0.21
350	34.0	34.4	33.1	33.8	0.44
360	33.8	34.1	33.1	33.7	0.26
370	33.8	33.8	32.9	33.5	0.27
380	33.4	33.7	32.8	33.3	0.21
390	33.2	33.4	32.5	33.0	0.22
400	32.8	33.3	32.3	32.8	0.25
410	32.6	33.1	32.1	32.6	0.25
420	32.5	32.8	31.9	32.4	0.21
430	32.2	32.7	31.7	32.2	0.25
440	31.9	32.6	31.5	32.0	0.31
450	31.6	32.4	31.3	31.8	0.32
460	31.4	32.1	31.1	31.5	0.26
470	31.4	32.1	31.1	31.5	0.26
480	31.1	31.9	30.8	31.3	0.32
490	31.0	31.7	30.6	31.1	0.31
500	30.6	31.7	30.3	30.9	0.54
510	30.4	31.2	30.3	30.6	0.24
520	30.2	31.0	30.0	30.4	0.28
530	30.2	30.8	29.7	30.2	0.30
540	30.0	30.7	29.6	30.1	0.31
550	29.7	30.6	29.3	29.9	0.44
560	29.7	30.2	29.3	29.7	0.20
Sample variance					0.33
				max D(T) =	1.60
				min D(T) =	0
				G=	0.0858
				Sum D(T)=	18.70
				G known=	0.1131

Table S8: Solvent variation, EtOH.

EtOH					
Time, s	T_1, °C	T_2, °C	T_3, °C	T_{med}, °C	D(T)

0	23.0	23.0	23.0	23.0	0.00
10	34.4	35.0	33.5	34.3	0.57
20	35.4	35.6	33.5	34.8	1.34
30	36.3	36.3	36.0	36.2	0.03
40	37.2	37.2	37.1	37.2	0.00
50	37.6	37.9	37.8	37.8	0.02
60	38.4	38.4	38.7	38.5	0.03
70	38.7	38.9	39.7	39.1	0.28
80	39.4	39.3	40.3	39.7	0.30
90	39.4	39.8	40.8	40.0	0.52
100	39.8	40.1	41.3	40.4	0.63
110	40.2	40.6	41.5	40.8	0.44
120	40.2	40.5	41.8	40.8	0.72
130	40.0	40.6	41.9	40.8	0.94
140	41.6	40.8	42.1	41.5	0.43
150	41.4	40.7	41.9	41.3	0.36
160	41.9	40.7	42.2	41.6	0.63
170	42.1	41.1	42.2	41.8	0.37
180	41.2	40.8	41.9	41.3	0.31
190	41.6	40.6	41.8	41.3	0.41
200	41.2	40.7	41.5	41.1	0.16
210	41.0	40.6	41.2	40.9	0.09
220	40.7	39.9	41.1	40.6	0.37
230	40.9	39.7	41.1	40.6	0.57
240	40.4	39.6	40.8	40.3	0.37
250	40.1	39.5	40.6	40.1	0.30
260	40.5	39.1	40.4	40.0	0.61
270	40.3	39.1	40.1	39.8	0.41
280	40.1	38.9	40.0	39.7	0.44
290	39.6	38.7	39.6	39.3	0.27
300	39.6	38.6	39.3	39.2	0.26
310	39.2	38.3	39.2	38.9	0.27
320	39.1	38.1	38.8	38.7	0.26
330	38.9	37.8	38.7	38.5	0.34
340	38.4	37.6	38.2	38.1	0.17
350	38.7	37.6	38.0	38.1	0.31
360	38.4	37.4	37.8	37.9	0.25
370	38.1	37.3	37.7	37.7	0.16
380	37.9	36.9	37.3	37.4	0.25
390	35.8	36.8	37.0	36.5	0.41
400	35.7	36.5	36.8	36.3	0.32
410	35.3	36.2	36.5	36.0	0.39
420	35.2	36.1	36.3	35.9	0.34
430	34.9	36.0	36.1	35.7	0.44
440	34.6	35.9	35.8	35.4	0.52

450	34.5	35.6	35.6	35.2	0.40
460	34.4	35.5	35.7	35.2	0.49
470	34.0	35.2	35.5	34.9	0.63
480	33.9	34.9	35.4	34.7	0.58
490	33.7	34.8	35.2	34.6	0.60
500	33.4	34.6	35.1	34.4	0.76
510	33.4	34.3	34.7	34.1	0.44
520	33.1	34.2	34.4	33.9	0.49
530	32.8	34.1	34.3	33.7	0.66
540	32.8	34.0	34.0	33.6	0.48
550	32.6	33.7	33.9	33.4	0.49
560	32.2	33.9	33.7	33.3	0.86
570	32.3	33.5	33.6	33.1	0.52
580	32.0	33.4	33.4	32.9	0.65
590	31.8	33.4	33.1	32.8	0.72
600	31.8	33.4	32.9	32.7	0.67
Sample variance					0.43
				max D(T) =	1.34
				min D(T) =	0
				G=	0.0513
				Sum D(T)=	26.18
				G known=	0.1131

Table S9: Solvent variation, iso-propanol.

i-PrOH					
Time, s	T_1, °C	T_2, °C	T_3, °C	T_{med}, °C	D(T)
0	23.0	23.0	23.0	23.0	0.00
10	29.3	32.1	24.9	28.8	13.17
20	32.8	34.4	30.0	32.4	4.96
30	32.1	36.4	29.3	32.6	12.79
40	35.4	37.9	30.3	34.5	15.00
50	37.3	38.9	34.1	36.8	5.97
60	38.7	40.1	38.5	39.1	0.76
70	40.1	41.3	37.1	39.5	4.68
80	41.1	42.4	39.7	41.1	1.82
90	42.0	43.1	41.4	42.2	0.74
100	42.2	43.7	42.4	42.8	0.66
110	42.9	43.9	43.3	43.4	0.25
120	42.9	44.0	43.7	43.5	0.32
130	43.2	44.0	44.0	43.7	0.21
140	43.6	44.5	44.7	44.3	0.34
150	43.5	44.7	44.3	44.2	0.37
160	43.6	44.3	44.1	44.0	0.13
170	43.3	44.2	44.1	43.9	0.24
180	43.3	44.0	43.6	43.6	0.12

190	42.9	43.8	43.6	43.4	0.22
200	42.9	43.6	43.2	43.2	0.12
210	42.7	43.7	42.8	43.1	0.30
220	42.2	43.4	42.4	42.7	0.41
230	41.9	43.2	41.7	42.3	0.66
240	41.6	42.8	41.4	41.9	0.57
250	41.3	42.4	40.8	41.5	0.67
260	40.9	42.0	40.3	41.1	0.74
270	40.9	41.8	40.0	40.9	0.81
280	40.4	42.2	39.6	40.7	1.77
290	40.1	41.4	39.2	40.2	1.22
300	39.9	41.0	38.8	39.9	1.21
310	39.5	40.8	38.4	39.6	1.44
320	39.4	40.5	38.0	39.3	1.57
330	39.0	40.3	37.6	39.0	1.82
340	38.7	39.8	37.3	38.6	1.57
350	38.5	39.6	37.0	38.4	1.70
360	38.2	39.7	36.6	38.2	2.40
370	37.9	39.3	36.3	37.8	2.25
380	37.7	39.2	36.1	37.7	2.40
390	37.6	38.8	35.7	37.4	2.44
400	37.2	38.5	35.4	37.0	2.42
410	37.1	38.2	34.9	36.7	2.82
420	36.8	38.0	34.9	36.6	2.44
430	36.6	37.5	34.4	36.2	2.54
440	36.2	37.3	34.1	35.9	2.64
450	35.9	37.4	33.8	35.7	3.27
460	35.6	37.2	33.6	35.5	3.25
470	35.3	36.9	33.6	35.3	2.72
480	35.4	36.6	33.1	35.0	3.16
490	35.0	36.5	32.9	34.8	3.27
500	35.0	36.1	32.7	34.6	3.01
510	34.7	35.7	32.5	34.3	2.68
520	34.4	35.6	32.4	34.1	2.61
530	34.3	35.3	32.0	33.9	2.86
540	34.0	37.6	32.0	34.5	8.05
550	33.8	37.3	31.8	34.3	7.75
560	33.7	36.9	31.6	34.1	7.12
570	33.4	37.0	31.7	34.0	7.32
580	33.4	36.8	31.4	33.9	7.45
590	33.4	36.7	31.2	33.8	7.66
600	33.4	36.5	31.0	33.6	7.60
Sample variance					2.98
				max D(T) =	15.00
				min D(T) =	0

G=	0.0826
Sum D(T)=	181.61
G known=	0.1131

Table S10: Solvent variation, dioxane.

Dioxane					
Time, s	T_1, °C	T_2, °C	T_3, °C	T_{med}, °C	D(T)
0	23.0	23.0	23.0	23.0	0.00
10	27.8	29.3	30.8	29.3	2.25
20	31.7	29.7	32.3	31.2	1.85
30	33.6	33.9	32.3	33.3	0.72
40	34.8	34.9	34.2	34.6	0.14
50	36.4	36.7	35.7	36.3	0.26
60	37.5	37.6	37.2	37.4	0.04
70	38.4	38.3	38.6	38.4	0.02
80	39.2	38.9	39.6	39.2	0.12
90	39.9	39.6	40.5	40.0	0.21
100	40.5	40.2	41.0	40.6	0.16
110	40.8	40.4	41.4	40.9	0.25
120	41.2	40.9	41.6	41.2	0.12
130	41.4	40.9	41.9	41.4	0.25
140	41.6	41.2	42.2	41.7	0.25
150	41.8	40.6	42.2	41.5	0.69
160	41.9	40.6	42.4	41.6	0.86
170	41.9	41.7	42.3	42.0	0.09
180	41.8	41.5	42.2	41.8	0.12
190	41.9	41.4	42.2	41.8	0.16
200	41.7	40.8	42.0	41.5	0.39
210	41.5	40.7	42.0	41.4	0.43
220	41.5	40.5	41.8	41.3	0.46
230	41.1	40.3	41.7	41.0	0.49
240	41.1	40.0	41.4	40.8	0.54
250	40.9	39.8	41.2	40.6	0.54
260	40.6	39.5	41.0	40.4	0.60
270	40.3	39.2	40.8	40.1	0.67
280	40.1	38.9	40.3	39.8	0.57
290	39.8	38.7	40.0	39.5	0.49
300	39.6	38.3	39.8	39.2	0.66
310	39.2	38.0	39.6	38.9	0.69
320	39.1	37.7	39.2	38.7	0.70
330	38.9	37.4	39.0	38.4	0.80
340	38.6	37.2	39.1	38.3	0.97
350	38.3	36.8	38.7	37.9	1.00
360	38.1	36.6	38.4	37.7	0.93

370	37.8	36.4	38.2	37.5	0.89
380	37.5	36.1	37.9	37.2	0.89
390	37.6	35.9	37.6	37.0	0.96
400	37.5	35.7	37.4	36.9	1.02
410	37.3	35.4	37.0	36.6	1.04
420	37.0	35.2	36.8	36.3	0.97
430	36.8	35.0	36.7	36.2	1.02
440	36.7	34.6	36.5	35.9	1.34
450	36.5	34.7	36.1	35.8	0.89
460	36.2	34.4	36.0	35.5	0.97
470	35.9	34.2	35.7	35.3	0.86
480	35.9	33.9	35.4	35.1	1.08
490	35.6	33.8	35.1	34.8	0.86
500	35.4	33.6	35.1	34.7	0.93
510	35.0	33.5	34.8	34.4	0.66
520	34.9	33.1	34.7	34.2	0.97
530	34.5	32.9	34.5	34.0	0.85
540	34.2	32.8	34.3	33.8	0.70
550	34.0	32.6	34.2	33.6	0.76
560	33.8	32.3	34.0	33.4	0.86
570	33.7	32.1	33.7	33.2	0.85
580	33.5	32.0	33.5	33.0	0.75
590	33.3	31.9	33.3	32.8	0.65
600	33.0	31.7	33.3	32.7	0.72
Sample variance					0.67
				max D(T) =	2.25
				min D(T) =	0
				G=	0.0547
				Sum D(T)=	41.14
				G known=	0.1131

Table S11: Solvent variation, dichloroethane.

DCE					
Time, s	T_1, °C	T_2, °C	T_3, °C	T_{med}, °C	D(T)
0	23.0	23.0	23.0	23.0	0.00
10	22.8	21.8	21.9	22.2	0.30
20	22.5	21.5	21.0	21.7	0.58
30	22.1	24.0	22.8	23.0	0.92
40	25.3	25.2	24.2	24.9	0.37
50	27.2	26.1	25.8	26.4	0.54
60	29.6	27.1	26.7	27.8	2.47
70	30.0	28.1	28.6	28.9	0.97
80	30.9	29.5	30.3	30.2	0.49
90	31.6	30.7	31.9	31.4	0.39

100	32.6	31.8	34.1	32.8	1.36
110	33.9	33.7	35.8	34.5	1.34
120	36.3	35.3	37.0	36.2	0.73
130	38.0	36.2	37.3	37.2	0.82
140	39.3	37.1	37.5	38.0	1.37
150	38.2	38.0	37.9	38.0	0.02
160	38.5	38.4	38.5	38.5	0.00
170	38.9	39.4	38.8	39.0	0.10
180	39.5	39.9	39.2	39.5	0.12
190	39.7	40.1	39.4	39.7	0.12
200	40.2	40.8	39.6	40.2	0.36
210	40.8	41.0	39.9	40.6	0.34
220	41.0	41.7	40.2	41.0	0.56
230	40.8	41.9	40.1	40.9	0.82
240	41.1	41.4	39.8	40.8	0.72
250	40.2	41.0	39.6	40.3	0.49
260	40.1	40.6	39.1	39.9	0.58
270	39.8	40.3	38.9	39.7	0.50
280	39.9	39.9	38.7	39.5	0.48
290	39.7	39.5	38.2	39.1	0.66
300	39.3	39.1	37.9	38.8	0.57
310	39.0	38.8	37.7	38.5	0.49
320	38.6	38.5	37.3	38.1	0.52
330	38.2	38.1	37.0	37.8	0.44
340	37.9	37.6	36.7	37.4	0.39
350	37.8	37.3	36.6	37.2	0.36
360	37.3	37.0	36.2	36.8	0.32
370	36.9	36.7	35.8	36.5	0.34
380	36.9	36.4	35.7	36.3	0.36
390	36.6	36.2	35.3	36.0	0.44
400	36.2	35.8	35.1	35.7	0.31
410	35.8	35.7	34.8	35.4	0.30
420	35.6	35.2	34.6	35.1	0.25
430	35.3	34.9	34.4	34.9	0.20
440	35.0	34.8	34.0	34.6	0.28
450	34.7	34.5	34.1	34.4	0.09
460	34.4	34.3	33.8	34.2	0.10
470	34.2	34.2	33.3	33.9	0.27
480	33.9	33.8	33.3	33.7	0.10
490	33.7	33.6	33.0	33.4	0.14
500	33.6	33.4	32.9	33.3	0.13
510	33.3	33.1	32.6	33.0	0.13
520	33.0	32.8	32.5	32.8	0.06
530	32.8	32.7	32.2	32.6	0.10
540	32.6	32.6	32.2	32.5	0.05

550	32.4	32.3	31.9	32.2	0.07
560	32.2	32.2	31.8	32.1	0.05
570	32.0	31.8	31.6	31.8	0.04
580	32.0	32.0	31.4	31.8	0.12
Sample variance					0.44
				max D(T) =	2.47
				min D(T) =	0
				G=	0.0962
				Sum D(T)=	25.68
				G known=	0.1131

Table S12: Solvent variation, hexane.

Hexane					
Time, s	T_1, °C	T_2, °C	T_3, °C	T_{med}, °C	D(T)
0	23.0	23.0	23.0	23.0	0.00
10	22.6	22.6	23.6	22.9	0.33
20	35.1	36.7	34.8	35.5	1.04
30	50.6	51.0	50.8	50.8	0.04
40	44.8	44.9	47.2	45.6	1.84
50	44.0	43.7	44.2	44.0	0.06
60	43.3	43.4	44.1	43.6	0.19
70	42.8	42.5	44.0	43.1	0.63
80	42.5	42.1	44.2	42.9	1.24
90	42.2	42.0	43.8	42.7	0.97
100	41.8	41.6	43.3	42.2	0.86
110	41.4	41.3	42.7	41.8	0.61
120	41.0	41.1	42.5	41.5	0.70
130	40.5	40.5	42.2	41.1	0.96
140	40.2	40.4	41.8	40.8	0.76
150	39.8	39.6	41.2	40.2	0.76
160	39.3	39.4	40.7	39.8	0.61
170	38.8	39.0	40.5	39.4	0.86
180	38.8	38.8	39.9	39.2	0.40
190	38.2	38.4	39.4	38.7	0.41
200	37.9	38.0	39.1	38.3	0.44
210	37.6	37.8	38.7	38.0	0.34
220	37.2	37.6	38.4	37.7	0.37
230	37.1	37.1	37.8	37.3	0.16
240	36.8	37.2	37.6	37.2	0.16
250	36.2	36.6	37.3	36.7	0.31
260	35.9	36.4	36.8	36.4	0.20
270	35.6	36.0	36.6	36.1	0.25
280	35.3	35.9	36.3	35.8	0.25
290	35.1	35.5	36.0	35.5	0.20

300	34.8	35.1	35.7	35.2	0.21
310	34.7	34.9	35.3	35.0	0.09
320	34.5	34.7	35.3	34.8	0.17
330	34.2	34.4	34.9	34.5	0.13
340	33.9	34.3	34.7	34.3	0.16
350	33.7	33.9	34.5	34.0	0.17
360	33.5	33.6	34.1	33.7	0.10
370	33.3	33.5	34.0	33.6	0.13
380	33.2	33.7	33.7	33.5	0.08
390	32.9	33.3	33.5	33.2	0.09
400	32.6	33.1	33.2	33.0	0.10
410	32.4	32.7	32.8	32.6	0.04
420	32.2	32.4	32.6	32.4	0.04
430	32.3	32.2	32.3	32.3	0.00
440	32.1	32.1	32.1	32.1	0.00
450	31.9	31.8	32.0	31.9	0.01
460	31.6	31.7	31.8	31.7	0.01
470	31.4	31.4	31.6	31.5	0.01
480	31.3	31.4	31.3	31.3	0.00
490	30.8	31.1	31.2	31.0	0.04
500	30.9	30.9	30.9	30.9	0.00
510	30.6	30.9	30.7	30.7	0.02
520	30.1	30.6	30.7	30.5	0.10
530	30.3	30.4	30.3	30.3	0.00
540	29.9	30.4	30.2	30.2	0.06
550	29.7	29.9	30.0	29.9	0.02
560	29.7	29.9	29.9	29.8	0.01
Sample variance					0.31
				max D(T) =	1.84
				min D(T) =	0
				G=	0.1032
				Sum D(T)=	17.87
				G known=	0.1131

Table S13: Solvent variation, ethylene glycol.

Ethylene glycol						
Time, s	T_1, °C	T_2, °C	T_3, °C	T_4, °C	T_{med}, °C	D(T)
0	23.0	23.0	23.0	23.0	23.0	0.00
10	28.9	31.6	28.1	28.6	29.3	2.46
20	28.3	30.1	27.9	28.1	28.6	1.03
30	28.4	29.4	28.0	27.5	28.3	0.65
40	30.5	28.7	30.2	27.2	29.2	2.31
50	31.5	30.1	30.7	29.0	30.3	1.11
60	32.0	30.5	31.0	29.6	30.8	1.00

70	31.9	30.5	31.1	29.6	30.8	0.94
80	31.7	30.8	29.0	30.1	30.4	1.30
90	32.0	31.0	29.0	30.2	30.6	1.61
100	32.0	30.9	29.4	30.4	30.7	1.17
110	32.1	31.2	29.5	30.4	30.8	1.23
120	32.3	31.2	29.7	30.5	30.9	1.22
130	32.1	31.5	29.4	30.6	30.9	1.38
140	32.4	31.5	29.7	30.8	31.1	1.30
150	32.6	31.4	29.8	30.7	31.1	1.40
160	32.7	31.1	29.9	30.7	31.1	1.39
170	32.7	31.2	29.9	30.8	31.2	1.36
180	32.7	31.3	29.9	30.9	31.2	1.35
190	32.7	31.4	30.0	30.9	31.3	1.27
200	32.7	31.6	30.0	33.1	31.9	1.92
210	31.1	31.4	31.4	33.2	31.8	0.92
220	31.2	31.5	31.5	32.9	31.8	0.58
230	31.1	31.7	31.4	32.9	31.8	0.62
240	31.2	31.7	31.5	33.0	31.9	0.63
250	31.3	31.7	31.8	33.2	32.0	0.69
260	31.1	31.6	31.7	33.1	31.9	0.74
270	31.2	30.9	31.6	33.1	31.7	0.95
280	31.2	31.1	31.4	33.2	31.7	0.98
290	31.2	30.9	31.5	33.1	31.7	0.96
300	31.2	31.0	31.7	33.3	31.8	1.09
310	31.3	31.0	31.6	33.0	31.7	0.78
320	31.2	30.9	31.6	32.9	31.7	0.78
330	31.1	31.1	31.5	32.9	31.7	0.73
340	31.2	30.9	31.3	32.8	31.6	0.72
350	31.2	31.0	31.2	32.9	31.6	0.79
360	31.2	31.0	31.2	32.9	31.6	0.79
370	31.2	30.9	31.2	32.8	31.5	0.74
380	31.3	31.0	31.2	32.9	31.6	0.77
390	31.2	30.9	31.2	32.8	31.5	0.74
400	31.2	31.4	31.3	32.7	31.7	0.50
410	31.1	31.5	31.4	32.8	31.7	0.57
420	31.1	31.4	31.1	32.7	31.6	0.58
430	31.1	31.3	31.1	32.6	31.5	0.52
440	31.3	31.5	30.9	34.9	32.2	3.42
450	31.2	31.2	31.1	34.0	31.9	2.01
460	30.9	31.2	31.6	33.0	31.7	0.86
470	30.7	31.3	31.5	32.7	31.6	0.70
480	30.7	31.3	31.4	32.8	31.6	0.79
490	30.7	31.5	31.4	32.6	31.6	0.62
500	30.5	31.3	31.4	32.8	31.5	0.91
510	30.6	31.4	31.6	32.9	31.6	0.91

520	30.6	31.3	31.3	32.9	31.5	0.95
530	30.5	31.0	31.4	32.8	31.4	0.98
540	30.6	31.0	31.5	32.5	31.4	0.67
550	30.6	31.0	31.5	32.6	31.4	0.75
560	30.6	30.9	31.4	32.7	31.4	0.86
570	30.7	31.1	31.3	30.4	30.9	0.16
580	30.7	30.9	31.4	30.3	30.8	0.21
590	30.5	30.9	31.4	30.2	30.8	0.27
600	30.4	30.9	31.6	30.1	30.8	0.43
Sample variance						0.98
					max D(T) =	3.42
					min D(T) =	0
					G=	0.0570
					Sum D(T)=	60.08
					G known=	0.0895

Table S14: Solvent variation, water.

H_2O					
Time, s	T_1, °C	T_2, °C	T_3, °C	T_{med}, °C	D(T)
0	23.0	23.0	23.0	23.0	0.00
10	23.3	22.7	22.3	22.8	0.25
20	59.2	54.8	56.9	57.0	4.84
30	71.3	69.4	73.8	71.5	4.87
40	57.7	57.0	58.3	57.7	0.42
50	48.2	49.6	50.3	49.4	1.14
60	44.5	42.3	44.9	43.9	1.96
70	41.7	39.9	42.5	41.4	1.77
80	39.4	38.1	38.5	38.7	0.44
90	37.7	36.6	38.3	37.5	0.74
100	36.0	35.5	38.3	36.6	2.23
110	34.7	34.6	38.3	35.9	4.44
120	33.7	33.6	37.7	35.0	5.47
130	33.5	33.6	37.6	34.9	5.47
140	33.5	33.7	37.7	35.0	5.61
150	33.3	33.5	37.6	34.8	5.89
160	33.1	33.4	37.3	34.6	5.49
170	33.0	33.2	37.0	34.4	5.08
180	33.1	33.1	37.2	34.5	5.60
190	33.0	33.0	37.0	34.3	5.33
200	32.9	32.9	36.9	34.2	5.33
210	32.9	32.9	36.8	34.2	5.07
220	32.8	32.8	36.7	34.1	5.07
230	32.6	32.6	36.6	33.9	5.33
240	32.5	32.5	36.4	33.8	5.07

250	32.6	32.6	36.3	33.8	4.56
260	32.3	32.3	36.1	33.6	4.81
270	32.2	32.2	36.0	33.5	4.81
280	32.2	32.2	35.9	33.4	4.56
290	32.1	32.1	35.8	33.3	4.56
300	31.9	31.9	35.7	33.2	4.81
310	32.0	32.0	35.7	33.2	4.56
320	31.6	31.6	35.5	32.9	5.07
330	31.5	31.5	35.4	32.8	5.07
340	31.5	31.5	35.1	32.7	4.32
350	31.3	31.3	35.1	32.6	4.81
360	31.2	31.2	35.1	32.5	5.07
370	31.2	31.2	35.0	32.5	4.81
380	31.2	31.2	34.9	32.4	4.56
390	31.0	31.0	34.7	32.2	4.56
400	31.0	31.0	34.5	32.2	4.08
410	30.8	30.8	34.5	32.0	4.56
420	30.6	30.6	34.2	31.8	4.32
430	30.6	30.6	34.0	31.7	3.85
440	30.4	30.4	34.0	31.6	4.32
450	30.4	30.4	34.0	31.6	4.32
460	30.4	30.4	33.8	31.5	3.85
470	30.3	30.3	33.8	31.5	4.08
480	30.3	30.3	33.8	31.5	4.08
490	30.0	30.0	33.9	31.3	5.07
500	30.1	30.1	33.7	31.3	4.32
510	29.9	29.9	33.7	31.2	4.81
520	30.0	30.0	33.3	31.1	3.63
530	30.0	30.0	33.2	31.1	3.41
540	29.8	29.8	33.1	30.9	3.63
550	29.7	29.7	32.9	30.8	3.41
560	29.7	29.7	32.7	30.7	3.00
570	29.3	29.3	32.6	30.4	3.63
580	29.4	29.4	32.6	30.5	3.41
590	29.3	29.3	32.7	30.4	3.85
600	29.2	29.2	32.6	30.3	3.85
Sample variance					4.06
				max D(T) =	5.89
				min D(T) =	0
				G=	0.0238
				Sum D(T)=	247.45
				G known=	0.1131

Table S15: Loading variations, 1.

0.87 g (0.65 g pure CaC_2)					
Time, s	T_1, °C	T_2, °C	T_3, °C	T_{med}, °C	D(T)
0	23.0	23.0	23.0	23.0	0.00
10	34.4	34.3	32.5	33.7	1.14
20	33.1	32.8	30.8	32.2	1.56
30	31.8	31.1	29.2	30.7	1.81
40	41.0	43.2	41.3	41.8	1.42
50	44.1	44.2	44.8	44.4	0.14
60	46.9	46.4	46.4	46.6	0.08
70	49.6	48.5	49.2	49.1	0.31
80	51.4	50.6	51.2	51.1	0.17
90	52.9	52.3	52.6	52.6	0.09
100	54.4	53.7	54.0	54.0	0.12
110	55.6	55.2	55.5	55.4	0.04
120	56.8	55.9	56.5	56.4	0.21
130	57.3	56.7	57.4	57.1	0.14
140	57.6	57.5	58.2	57.8	0.14
150	57.9	57.9	58.9	58.2	0.33
160	58.2	58.3	59.5	58.7	0.52
170	58.1	58.5	59.4	58.7	0.44
180	58.2	58.5	59.8	58.8	0.72
190	58.1	58.6	60.0	58.9	0.97
200	58.0	58.4	59.9	58.8	1.00
210	57.7	58.3	59.8	58.6	1.17
220	57.5	58.0	59.6	58.4	1.20
230	57.1	57.7	59.3	58.0	1.29
240	56.7	57.5	59.2	57.8	1.63
250	56.4	57.1	58.9	57.5	1.66
260	56.1	56.8	58.6	57.2	1.66
270	55.6	56.5	57.9	56.7	1.34
280	55.2	56.2	57.8	56.4	1.72
290	54.6	55.7	57.3	55.9	1.84
300	54.3	55.4	57.0	55.6	1.84
310	53.9	55.0	56.4	55.1	1.57
320	53.3	54.4	56.2	54.6	2.14
330	52.9	54.0	55.6	54.2	1.84
340	52.6	53.6	55.3	53.8	1.86
350	52.1	53.2	54.8	53.4	1.84
360	51.7	52.9	54.5	53.0	1.97
370	51.1	52.3	54.2	52.5	2.44
380	50.8	52.0	53.5	52.1	1.83
390	50.5	51.7	53.1	51.8	1.69
400	50.0	51.1	52.8	51.3	1.99
410	49.8	50.7	52.4	51.0	1.74
420	49.3	50.3	51.8	50.5	1.58

430	48.9	50.1	51.6	50.2	1.83
440	48.4	49.9	51.1	49.8	1.83
450	48.1	49.4	50.9	49.5	1.96
460	47.7	49.5	50.5	49.2	2.01
470	47.2	48.9	50.1	48.7	2.12
480	46.8	48.4	49.6	48.3	1.97
490	46.5	47.9	49.2	47.9	1.82
500	46.2	47.5	48.8	47.5	1.69
510	45.8	46.6	48.3	46.9	1.63
520	45.3	46.4	47.9	46.5	1.70
530	44.8	46.0	47.5	46.1	1.83
540	44.6	45.5	47.0	45.7	1.47
550	44.0	45.4	46.7	45.4	1.82
Sample variance					1.30
				max D(T) =	2.44
				min D(T) =	0
				G=	0.0335
				Sum D(T)=	73.00
				G known=	0.1131

Table S16: Loading variations, 2.

1.28 g (0.96 g pure CaC_2)					
Time, s	T_1, °C	T_2, °C	T_3, °C	T_{med}, °C	D(T)
0	23.0	23.0	23.0	23.0	0.00
10	34.3	31.9	34.6	33.6	2.19
20	33.8	30.7	33.2	32.6	2.70
30	45.1	44.0	42.5	43.9	1.70
40	49.6	48.1	48.9	48.9	0.56
50	53.7	52.2	53.2	53.0	0.58
60	57.8	55.5	57.5	56.9	1.56
70	61.3	58.9	61.2	60.5	1.84
80	64.3	61.4	64.0	63.2	2.54
90	66.6	64.0	67.0	65.9	2.65
100	68.8	66.0	69.4	68.1	3.29
110	70.4	67.8	70.8	69.7	2.65
120	71.6	69.0	71.9	70.8	2.54
130	72.2	69.9	72.9	71.7	2.46
140	72.8	70.6	73.5	72.3	2.29
150	73.1	71.1	73.8	72.7	1.96
160	72.8	71.4	74.0	72.7	1.69
170	72.6	71.4	73.9	72.6	1.56
180	72.6	71.7	73.4	72.6	0.72
190	72.1	71.2	73.4	72.2	1.22
200	71.7	71.1	73.1	72.0	1.05
210	71.2	70.7	72.5	71.5	0.86

220	70.5	70.3	72.0	70.9	0.86
230	69.9	69.7	71.6	70.4	1.09
240	69.4	69.4	71.1	70.0	0.96
250	68.7	68.7	70.4	69.3	0.96
260	68.0	68.2	70.0	68.7	1.21
270	66.9	67.5	69.4	67.9	1.70
280	66.4	66.9	69.0	67.4	1.90
290	65.5	66.1	68.4	66.7	2.34
300	65.2	65.6	67.8	66.2	1.96
310	64.7	64.8	66.9	65.5	1.54
320	64.1	64.2	66.4	64.9	1.69
330	63.6	63.6	65.7	64.3	1.47
340	63.2	63.1	65.0	63.8	1.14
350	62.7	62.4	64.4	63.2	1.16
360	62.2	61.7	63.9	62.6	1.33
370	61.5	61.1	63.3	62.0	1.37
380	61.0	60.6	62.8	61.5	1.37
390	60.6	60.0	62.0	60.9	1.05
400	60.0	59.7	61.5	60.4	0.93
410	59.7	58.9	61.0	59.9	1.12
420	59.1	58.5	60.6	59.4	1.17
430	58.5	57.9	59.8	58.7	0.94
440	58.0	57.5	59.3	58.3	0.86
450	57.0	56.7	58.4	57.4	0.82
460	56.5	56.3	57.9	56.9	0.76
470	56.0	55.7	57.4	56.4	0.82
480	55.4	55.0	57.0	55.8	1.12
490	54.7	54.4	56.6	55.2	1.42
500	54.3	54.0	56.1	54.8	1.29
510	53.9	53.6	55.7	54.4	1.29
520	53.3	53.0	55.1	53.8	1.29
530	52.9	52.5	55.7	53.7	3.04
540	52.4	52.2	55.1	53.2	2.62
550	51.8	51.7	55.1	52.9	3.74
Sample variance					1.55
				max D(T) =	3.74
				min D(T) =	0
				G=	0.0430
				Sum D(T)=	87.07
				G known=	0.1131

Table S17: Loading variations, 3.

1.76 g (1.32 g pure CaC_2)					
Time, s	T_1, °C	T_2, °C	T_3, °C	T_{med}, °C	D(T)
0	23.0	23.0	23.0	23.0	0.00

10	33.2	34.4	34.4	34.0	0.48
20	32.3	34.3	33.3	33.3	1.00
30	34.9	38.2	36.9	36.7	2.76
40	48.7	49.2	50.2	49.4	0.58
50	56.4	57.7	58.2	57.4	0.86
60	62.8	62.6	64.6	63.3	1.21
70	66.7	67.4	70.1	68.1	3.22
80	72.3	71.1	73.6	72.3	1.56
90	75.9	75.0	77.2	76.0	1.22
100	78.6	77.9	81.1	79.2	2.83
110	81.1	80.3	82.6	81.3	1.36
120	82.3	81.6	83.3	82.4	0.73
130	83.6	83.2	84.1	83.6	0.20
140	83.6	83.8	84.1	83.8	0.06
150	84.2	84.3	83.7	84.1	0.10
160	83.6	84.3	83.6	83.8	0.16
170	83.5	84.2	82.9	83.5	0.42
180	82.8	84.1	82.2	83.0	0.94
190	82.1	83.2	81.7	82.3	0.60
200	82.0	82.7	80.8	81.8	0.92
210	80.4	82.1	80.2	80.9	1.09
220	82.2	81.3	79.4	81.0	2.04
230	82.1	80.5	78.1	80.2	4.05
240	82.0	79.8	77.2	79.7	5.77
250	81.7	78.8	76.2	78.9	7.57
260	79.5	78.1	75.4	77.7	4.34
270	79.6	77.3	74.6	77.2	6.26
280	80.1	76.8	74.5	77.1	7.92
290	78.9	75.9	73.1	76.0	8.41
300	78.6	74.8	72.3	75.2	10.06
310	78.1	73.7	71.5	74.4	11.29
320	76.6	72.8	70.7	73.4	8.94
330	76.2	72.3	69.7	72.7	10.70
340	75.6	71.4	68.8	71.9	11.77
350	71.1	70.4	68.2	69.9	2.29
360	70.5	70.0	67.5	69.3	2.58
370	69.7	68.9	66.7	68.4	2.41
380	69.2	68.3	65.8	67.8	3.10
390	68.1	67.4	65.0	66.8	2.64
400	67.1	66.5	64.2	65.9	2.34
410	66.4	65.9	63.4	65.2	2.58
420	65.8	64.8	63.0	64.5	2.01
430	65.5	64.3	62.3	64.0	2.61
440	64.6	63.3	61.5	63.1	2.42
450	64.0	62.5	61.0	62.5	2.25

460	63.3	62.0	60.4	61.9	2.11
470	62.8	61.1	59.8	61.2	2.26
480	62.0	60.6	59.2	60.6	1.96
490	61.5	60.0	58.9	60.1	1.70
500	60.8	59.4	58.5	59.6	1.34
510	60.3	58.4	57.4	58.7	2.17
520	59.7	58.1	56.9	58.2	1.97
530	59.0	57.4	56.5	57.6	1.60
540	58.5	56.8	55.7	57.0	1.99
550	58.0	56.2	55.1	56.4	2.14
Sample variance					3.00
				max D(T) =	11.77
				min D(T) =	0
				G=	0.0701
				Sum D(T)=	168.03
				G known=	0.1131

Table S18: Ratio variations, 2.5 mL DMSO + 3.5 mL H2O

Time, s	T_1, °C	T_2, °C	T_3, °C	T_{med}, °C	D(T)
0	23.0	23.0	23.0	23.0	0.00
10	30.4	31.1	30.9	30.8	0.13
20	35.3	36.2	35.6	35.7	0.21
30	34.3	32.0	33.6	33.3	1.39
40	32.7	34.0	32.2	33.0	0.86
50	31.8	33.3	31.0	32.0	1.36
60	31.4	31.9	34.2	32.5	2.23
70	35.6	36.5	37.9	36.7	1.34
80	58.1	58.4	59.9	58.8	0.93
90	62.3	59.3	60.3	60.6	2.33
100	59.0	58.8	58.9	58.9	0.01
110	55.7	55.7	57.5	56.3	1.08
120	52.2	52.8	55.2	53.4	2.52
130	49.9	50.0	53.1	51.0	3.31
140	49.3	48.8	51.4	49.8	1.90
150	49.5	49.4	50.0	49.6	0.10
160	49.5	49.1	49.7	49.4	0.09
170	49.5	48.7	49.7	49.3	0.28
180	49.3	48.2	49.0	48.8	0.32
190	48.8	48.2	48.4	48.5	0.09
200	48.5	48.0	47.9	48.1	0.10
210	48.1	47.6	47.5	47.7	0.10
220	47.5	46.8	48.0	47.4	0.36
230	47.3	46.6	47.6	47.2	0.26

240	46.8	46.1	46.9	46.6	0.19
250	46.4	45.7	46.4	46.2	0.16
260	45.8	45.5	46.2	45.8	0.12
270	45.5	44.9	45.5	45.3	0.12
280	45.1	44.7	45.7	45.2	0.25
290	44.6	44.4	45.4	44.8	0.28
300	44.2	44.1	44.9	44.4	0.19
310	43.8	43.5	44.7	44.0	0.39
320	43.4	43.3	44.2	43.6	0.24
330	43.0	42.8	43.7	43.2	0.22
340	42.7	42.5	43.4	42.9	0.22
350	42.6	42.4	43.0	42.7	0.09
360	42.1	41.9	43.0	42.3	0.34
370	41.8	41.5	42.5	41.9	0.26
380	41.5	41.1	42.3	41.6	0.37
390	41.1	40.8	41.8	41.2	0.26
400	40.9	40.5	41.5	41.0	0.25
410	40.8	40.3	41.1	40.7	0.16
420	40.3	39.9	40.7	40.3	0.16
430	40.3	39.3	41.0	40.2	0.73
440	39.9	39.4	40.7	40.0	0.43
450	39.4	39.1	40.5	39.7	0.54
460	39.1	38.8	40.3	39.4	0.63
470	38.9	38.6	39.8	39.1	0.39
480	38.8	38.3	39.5	38.9	0.36
490	38.5	38.0	39.1	38.5	0.30
500	38.3	38.0	38.8	38.4	0.16
510	38.2	37.6	39.0	38.3	0.49
520	37.6	37.4	38.7	37.9	0.49
530	37.5	37.3	38.5	37.8	0.41
540	37.3	37.0	38.2	37.5	0.39
550	37.2	36.7	38.1	37.3	0.50
560	36.9	36.6	37.8	37.1	0.39
570	36.7	36.4	37.5	36.9	0.32
Sample variance					0.56
				max D(T) =	3.31
				min D(T) =	0
				G=	0.1028
				Sum D(T)=	32.21
				G known=	0.1131

Table S19: Ratio variations, 3 mL DMSO + 3 mL H_2O

3 mL DMSO + 3 mL H_2O					
Time, s	T_1, °C	T_2, °C	T_3, °C	T_{med}, °C	D(T)

0	23.0	23.0	23.0	23.0	0.00
10	28.9	29.6	29.0	29.2	0.14
20	27.5	27.4	28.6	27.8	0.44
30	36.0	40.1	37.3	37.8	4.39
40	34.7	37.6	37.7	36.7	2.90
50	31.8	36.1	35.2	34.4	5.14
60	30.8	33.9	33.3	32.7	2.70
70	41.3	42.8	40.1	41.4	1.83
80	50.3	50.7	54.3	51.8	4.85
90	55.4	55.5	56.4	55.8	0.30
100	58.8	58.8	58.4	58.7	0.05
110	60.0	58.8	57.4	58.7	1.69
120	56.9	57.6	54.5	56.3	2.64
130	53.8	56.2	53.3	54.4	2.40
140	51.3	54.7	51.5	52.5	3.64
150	49.8	53.1	51.7	51.5	2.74
160	49.9	51.2	51.2	50.8	0.56
170	49.5	50.6	51.1	50.4	0.67
180	49.5	50.8	51.2	50.5	0.79
190	47.9	50.6	51.2	49.9	3.09
200	47.6	50.2	50.3	49.4	2.34
210	48.4	49.9	50.1	49.5	0.86
220	48.2	49.4	49.7	49.1	0.63
230	47.8	49.3	49.2	48.8	0.70
240	47.0	48.8	48.8	48.2	1.08
250	46.9	48.7	48.3	48.0	0.89
260	46.8	48.3	47.8	47.6	0.58
270	46.0	47.7	47.3	47.0	0.79
280	46.6	47.2	46.9	46.9	0.09
290	44.8	46.8	46.9	46.2	1.40
300	44.8	46.2	46.2	45.7	0.65
310	44.7	46.0	45.5	45.4	0.43
320	44.0	45.8	45.1	45.0	0.82
330	44.5	45.2	44.7	44.8	0.13
340	43.7	44.6	44.4	44.2	0.22
350	43.5	44.1	44.3	44.0	0.17
360	43.5	43.9	43.8	43.7	0.04
370	43.0	43.6	43.5	43.4	0.10
380	42.8	43.1	42.7	42.9	0.04
390	42.3	42.8	42.6	42.6	0.06
400	42.2	42.4	42.3	42.3	0.01
410	41.8	42.0	42.0	41.9	0.01
420	42.0	41.6	41.5	41.7	0.07
430	41.9	41.3	41.2	41.5	0.14
440	41.0	40.9	40.6	40.8	0.04

450	40.7	40.7	40.4	40.6	0.03
460	40.5	40.3	40.0	40.3	0.06
470	40.2	39.9	39.4	39.8	0.16
480	40.2	39.9	39.1	39.7	0.32
490	39.4	39.4	38.9	39.2	0.08
500	39.3	39.2	38.5	39.0	0.19
510	39.0	38.8	38.5	38.8	0.06
520	38.7	38.8	38.0	38.5	0.19
530	38.8	38.3	37.9	38.3	0.20
540	38.3	38.1	37.6	38.0	0.13
550	38.4	37.9	37.3	37.9	0.30
560	38.4	37.6	37.0	37.7	0.49
570	37.1	37.4	36.8	37.1	0.09
580	37.0	37.1	36.6	36.9	0.07
590	36.6	37.1	36.3	36.7	0.16
600	36.5	37.1	36.3	36.6	0.17
Sample variance					0.92
				max D(T) =	5.14
				min D(T) =	0
				G=	0.0917
				Sum D(T)=	56.08
				G known=	0.1131

Table S20: Ratio variations, 3.5 mL DMSO + 2.5 mL H_2O

3.5 mL DMSO + 2.5 mL H_2O					
Time, s	T_1, °C	T_2, °C	T_3, °C	T_{med}, °C	D(T)
0	23.0	23.0	23.0	23.0	0.00
10	29.4	29.6	29.4	29.5	0.01
20	33.3	33.4	33.3	33.3	0.00
30	33.3	34.1	32.3	33.2	0.81
40	33.1	33.5	34.9	33.8	0.89
50	36.8	36.6	37.5	37.0	0.22
60	43.9	43.3	43.5	43.6	0.09
70	46.5	45.9	47.9	46.8	1.05
80	49.2	49.7	50.6	49.8	0.50
90	50.4	50.6	50.9	50.6	0.06
100	50.8	51.1	51.7	51.2	0.21
110	51.1	51.5	52.2	51.6	0.31
120	51.5	51.9	52.2	51.9	0.12
130	51.3	51.9	52.8	52.0	0.57
140	51.4	52.1	52.4	52.0	0.26
150	51.4	52.0	52.4	51.9	0.25
160	51.4	51.9	52.0	51.8	0.10
170	51.5	51.6	51.9	51.7	0.04

180	51.3	51.6	51.6	51.5	0.03
190	51.2	51.1	51.2	51.2	0.00
200	50.7	51.0	51.1	50.9	0.04
210	49.8	50.6	50.6	50.3	0.21
220	49.8	50.2	50.1	50.0	0.04
230	49.5	49.8	49.8	49.7	0.03
240	48.7	49.5	49.3	49.2	0.17
250	48.7	49.2	48.9	48.9	0.06
260	47.9	48.7	48.4	48.3	0.16
270	47.6	48.2	48.2	48.0	0.12
280	47.3	47.6	47.4	47.4	0.02
290	46.5	47.2	47.0	46.9	0.13
300	46.2	46.7	46.5	46.5	0.06
310	45.8	46.2	46.2	46.1	0.05
320	45.3	45.8	45.7	45.6	0.07
330	45.1	45.5	45.3	45.3	0.04
340	44.5	45.0	44.9	44.8	0.07
350	44.0	44.7	44.5	44.4	0.13
360	43.7	44.2	44.1	44.0	0.07
370	43.7	43.7	43.9	43.8	0.01
380	43.1	43.4	43.1	43.2	0.03
390	42.7	43.0	43.0	42.9	0.03
400	42.2	42.9	42.4	42.5	0.13
410	41.9	42.5	41.9	42.1	0.12
420	41.4	42.3	41.6	41.8	0.22
430	41.2	41.7	41.4	41.4	0.06
440	41.1	41.6	40.9	41.2	0.13
450	40.5	41.5	40.6	40.9	0.30
460	40.3	41.1	40.5	40.6	0.17
470	39.8	40.7	40.0	40.2	0.22
480	39.5	40.1	39.5	39.7	0.12
490	39.1	39.8	39.3	39.4	0.13
500	39.1	39.6	39.0	39.2	0.10
510	38.8	39.1	38.6	38.8	0.06
520	38.5	38.9	38.3	38.6	0.09
530	38.5	38.4	38.0	38.3	0.07
540	38.8	38.2	37.8	38.3	0.25
550	38.5	37.9	37.7	38.0	0.17
Sample variance					0.17
				max D(T) =	1.05
				min D(T) =	0
				G=	0.1107
				Sum D(T)=	9.52
				G known=	0.1131

Table S21: Ratio variations, 4.5 mL DMSO + 1.5 mL H_2O

4.5 mL DMSO + 1.5 mL H_2O					
Time, s	T_1, °C	T_2, °C	T_3, °C	T_{med}, °C	D(T)
0	23.0	23.0	23.0	23.0	0.00
10	28.2	28.8	29.6	28.9	0.49
20	34.9	34.6	33.4	34.3	0.63
30	35.7	34.8	35.4	35.3	0.21
40	33.0	33.5	34.4	33.6	0.50
50	39.0	38.4	37.9	38.4	0.30
60	40.8	40.1	40.2	40.4	0.14
70	41.7	41.0	41.1	41.3	0.14
80	42.2	41.8	42.3	42.1	0.07
90	42.9	42.4	43.2	42.8	0.16
100	43.7	43.1	43.9	43.6	0.17
110	44.4	44.1	44.9	44.5	0.16
120	44.9	44.5	45.6	45.0	0.31
130	45.5	45.3	46.3	45.7	0.28
140	45.9	45.7	47.2	46.3	0.66
150	46.4	45.8	47.7	46.6	0.94
160	46.6	46.2	47.9	46.9	0.79
170	47.1	46.8	48.5	47.5	0.82
180	47.0	46.8	48.6	47.5	0.97
190	47.4	47.0	49.0	47.8	1.12
200	47.7	47.4	49.2	48.1	0.93
210	47.5	47.1	49.1	47.9	1.12
220	47.5	47.1	49.0	47.9	1.00
230	47.5	47.2	49.1	47.9	1.04
240	47.4	47.1	48.9	47.8	0.93
250	47.5	47.2	48.7	47.8	0.63
260	47.3	46.9	48.7	47.6	0.89
270	47.1	46.9	48.8	47.6	1.09
280	46.9	46.6	48.5	47.3	1.04
290	46.8	46.8	48.3	47.3	0.75
300	46.6	46.5	47.8	47.0	0.52
310	46.5	46.3	47.4	46.7	0.34
320	46.3	46.0	47.2	46.5	0.39
330	45.9	45.6	46.9	46.1	0.46
340	45.8	45.3	46.4	45.8	0.30
350	45.6	45.3	46.0	45.6	0.12
360	45.1	44.8	45.9	45.3	0.32
370	44.2	44.5	45.4	44.7	0.39
380	44.1	44.2	45.0	44.4	0.24
390	43.8	44.0	44.5	44.1	0.13
400	43.5	43.8	44.4	43.9	0.21
410	43.0	43.5	44.2	43.6	0.36

420	42.8	43.2	43.6	43.2	0.16
430	42.7	43.0	43.1	42.9	0.04
440	42.3	42.8	42.8	42.6	0.08
450	42.2	42.3	42.6	42.4	0.04
460	41.9	41.9	42.2	42.0	0.03
470	41.6	41.7	41.9	41.7	0.02
480	41.2	41.6	41.5	41.4	0.04
490	40.8	41.1	41.3	41.1	0.06
500	40.8	41.0	40.8	40.9	0.01
510	40.4	40.6	40.5	40.5	0.01
520	40.0	40.3	40.0	40.1	0.03
530	39.8	39.8	39.6	39.7	0.01
540	39.6	39.5	39.3	39.5	0.02
550	39.5	39.2	39.2	39.3	0.03
560	39.2	38.8	38.9	39.0	0.04
570	39.2	38.7	38.5	38.8	0.13
580	39.5	38.7	38.6	38.9	0.24
590	39.2	38.5	37.9	38.5	0.42
Sample variance					0.39
				max D(T) =	1.12
				min D(T) =	0
				G=	0.0475
				Sum D(T)=	23.59
				G known=	0.1131

Table S22: Ratio variations, 5 mL DMSO + 1 mL H_2O

5 mL DMSO + 1 mL H_2O					
Time, s	T_1, °C	T_2, °C	T_3, °C	T_{med}, °C	D(T)
0	23.0	23.0	23.0	23.0	0.00
10	27.5	23.3	28.7	26.5	8.04
20	26.8	23.2	23.4	24.5	4.09
30	27.7	26.2	27.7	27.2	0.75
40	31.4	26.1	29.4	29.0	7.16
50	29.5	31.7	33.0	31.4	3.13
60	32.4	31.1	32.1	31.9	0.46
70	35.8	34.6	33.3	34.6	1.56
80	36.4	35.2	34.5	35.4	0.92
90	37.0	35.7	34.7	35.8	1.33
100	37.7	36.2	35.1	36.3	1.70
110	38.3	36.8	35.6	36.9	1.83
120	38.6	37.5	36.2	37.4	1.44
130	39.1	37.9	36.6	37.9	1.56
140	39.4	38.0	37.1	38.2	1.34
150	40.1	38.4	37.5	38.7	1.74

160	40.4	38.8	37.6	38.9	1.97
170	40.5	39.1	37.9	39.2	1.69
180	40.8	39.1	38.3	39.4	1.63
190	40.9	39.4	38.4	39.6	1.58
200	41.3	40.0	38.6	40.0	1.82
210	41.5	39.7	38.9	40.0	1.77
220	41.7	40.0	38.9	40.2	1.99
230	41.8	39.8	39.0	40.2	2.08
240	42.0	40.3	39.2	40.5	1.99
250	42.0	40.1	39.2	40.4	2.04
260	42.0	40.3	39.4	40.6	1.74
270	41.9	40.2	39.2	40.4	1.86
280	41.8	40.3	39.3	40.5	1.58
290	41.6	40.3	39.4	40.4	1.22
300	41.6	40.6	39.5	40.6	1.10
310	41.6	40.5	39.8	40.6	0.82
320	41.8	40.3	39.4	40.5	1.47
330	41.6	40.6	39.6	40.6	1.00
340	41.5	40.5	39.7	40.6	0.81
350	41.8	40.5	39.5	40.6	1.33
360	41.5	40.7	39.7	40.6	0.81
370	41.4	40.2	39.5	40.4	0.92
380	41.2	40.4	39.3	40.3	0.91
390	41.1	40.1	39.1	40.1	1.00
400	40.8	40.2	39.1	40.0	0.74
410	40.8	40.2	38.9	40.0	0.94
420	40.9	40.0	38.9	39.9	1.00
430	40.8	39.7	38.9	39.8	0.91
440	40.9	39.6	39.0	39.8	0.94
450	40.5	39.4	38.8	39.6	0.74
460	40.6	39.6	38.8	39.7	0.81
470	40.1	39.4	38.7	39.4	0.49
480	40.3	39.3	38.7	39.4	0.65
490	40.0	39.1	38.4	39.2	0.64
500	39.9	38.8	38.4	39.0	0.60
510	39.6	38.9	38.2	38.9	0.49
520	39.4	38.9	38.1	38.8	0.43
530	39.5	38.6	37.8	38.6	0.72
540	39.1	38.6	37.8	38.5	0.43
550	39.2	38.3	37.7	38.4	0.57
560	39.2	38.4	37.6	38.4	0.64
570	38.9	38.1	37.4	38.1	0.56
580	38.7	38.0	37.3	38.0	0.49
590	38.6	37.8	37.1	37.8	0.56
600	38.3	37.6	36.8	37.6	0.56

Sample variance	1.41
max D(T) =	8.04
min D(T) =	0
G=	0.0933
Sum D(T)=	86.22
G known=	0.1131

Table S23: Ratio variations, 5.5 mL DMSO + 0.5 mL H_2O

5.5 mL DMSO + 0.5 mL H_2O					
Time, s	T_1, °C	T_2, °C	T_3, °C	T_{med}, °C	D(T)
0	23.0	23.0	23.0	23.0	0.00
10	30.6	29.3	30.2	30.0	0.44
20	36.1	35.3	36.4	35.9	0.32
30	34.9	35.0	36.0	35.3	0.37
40	33.1	34.0	34.4	33.8	0.44
50	30.6	30.8	31.6	31.0	0.28
60	29.8	30.2	30.7	30.2	0.20
70	29.9	29.5	29.8	29.7	0.04
80	29.8	29.9	29.6	29.8	0.02
90	30.0	29.8	29.9	29.9	0.01
100	30.0	30.2	30.1	30.1	0.01
110	30.1	30.3	30.2	30.2	0.01
120	30.2	30.5	30.3	30.3	0.02
130	30.6	30.6	30.6	30.6	0.00
140	30.6	30.7	30.3	30.5	0.04
150	30.7	30.9	30.4	30.7	0.06
160	30.7	30.9	30.5	30.7	0.04
170	31.3	31.1	30.9	31.1	0.04
180	31.1	31.2	31.0	31.1	0.01
190	31.3	31.4	31.0	31.2	0.04
200	31.2	31.5	31.2	31.3	0.03
210	31.4	31.5	31.3	31.4	0.01
220	31.4	31.6	31.1	31.4	0.06
230	31.4	31.5	31.3	31.4	0.01
240	31.5	31.6	31.3	31.5	0.02
250	31.5	31.7	31.5	31.6	0.01
260	31.8	31.4	31.4	31.5	0.05
270	31.8	31.6	31.5	31.6	0.02
280	31.8	32.1	31.6	31.8	0.06
290	31.6	32.1	31.7	31.8	0.07
300	31.6	32.3	31.7	31.9	0.14
310	31.5	32.1	31.8	31.8	0.09
320	31.6	32.2	31.9	31.9	0.09
330	31.6	32.1	31.9	31.9	0.06

340	32.6	32.2	32.1	32.3	0.07
350	32.8	32.3	31.9	32.3	0.20
360	33.2	32.3	32.1	32.5	0.34
370	33.1	32.2	32.1	32.5	0.30
380	33.3	32.3	32.1	32.6	0.41
390	33.4	32.4	32.1	32.6	0.46
400	33.5	32.4	32.0	32.6	0.60
410	33.6	32.4	32.0	32.7	0.69
420	33.4	32.5	32.2	32.7	0.39
430	33.4	32.0	32.1	32.5	0.61
440	33.7	32.1	32.1	32.6	0.85
450	33.4	32.1	32.0	32.5	0.61
460	33.6	32.1	32.1	32.6	0.75
470	33.7	32.2	32.0	32.6	0.86
480	33.4	32.0	32.1	32.5	0.61
490	33.4	32.0	32.1	32.5	0.61
500	33.6	32.0	32.0	32.5	0.85
510	33.5	32.1	32.1	32.6	0.65
520	33.4	32.2	32.0	32.5	0.57
530	33.5	32.1	32.0	32.5	0.70
540	33.4	32.0	32.1	32.5	0.61
550	33.6	32.2	31.9	32.6	0.82
560	33.6	31.9	31.9	32.5	0.96
570	33.7	31.9	31.9	32.5	1.08
580	33.5	31.8	32.3	32.5	0.76
590	31.9	31.8	31.9	31.9	0.00
Sample variance					0.31
				max D(T) =	1.08
				min D(T) =	0
				G=	0.0581
				Sum D(T)=	18.59
				G known=	0.1131

6. NMR data

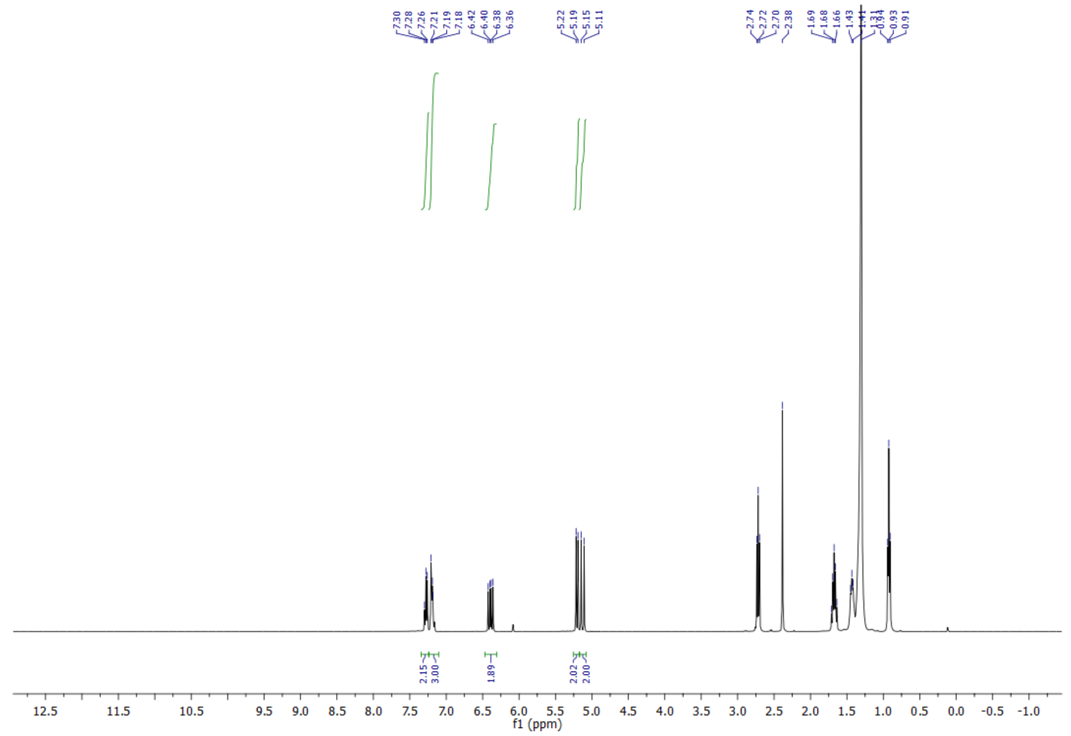

Figure S4: ^{1}H NMR spectrum of the reaction mixture after synthesis in a tube-in-tube reactor (toluene as an internal standard). The signals of toluene aromatic protons were chosen as a standard and were compared with signals of thiovinyl ether -CH=CH2 protons.

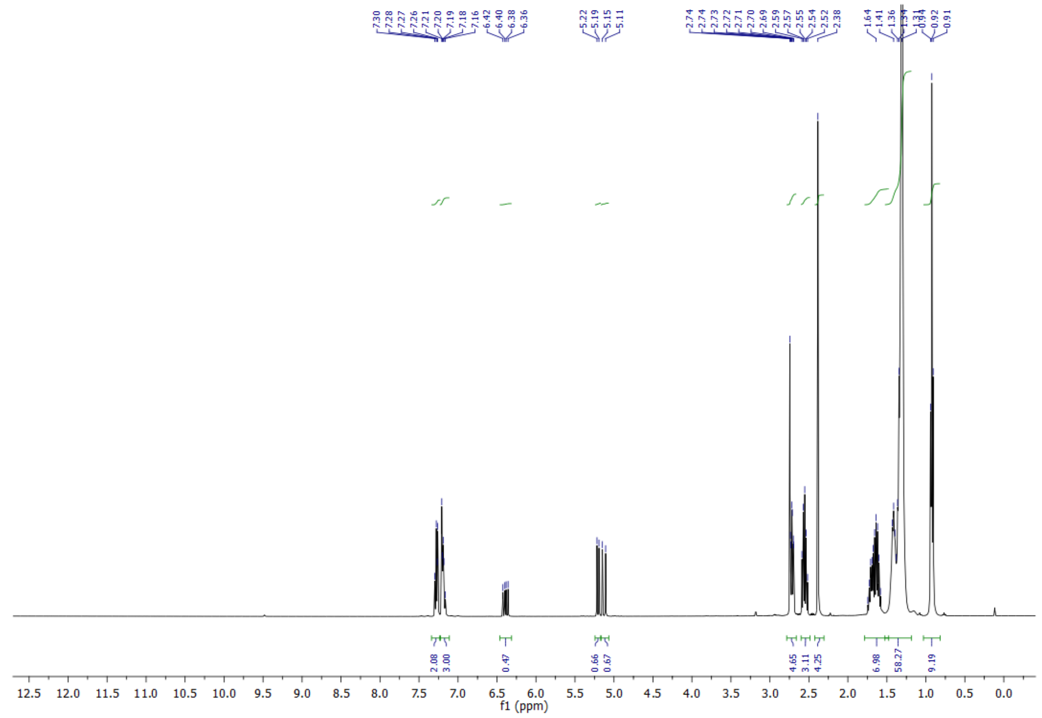

Figure S5: ^{1}H NMR spectrum of the reaction mixture after synthesis in a two-chamber reactor (toluene as an internal standard).

7. Reaction setup

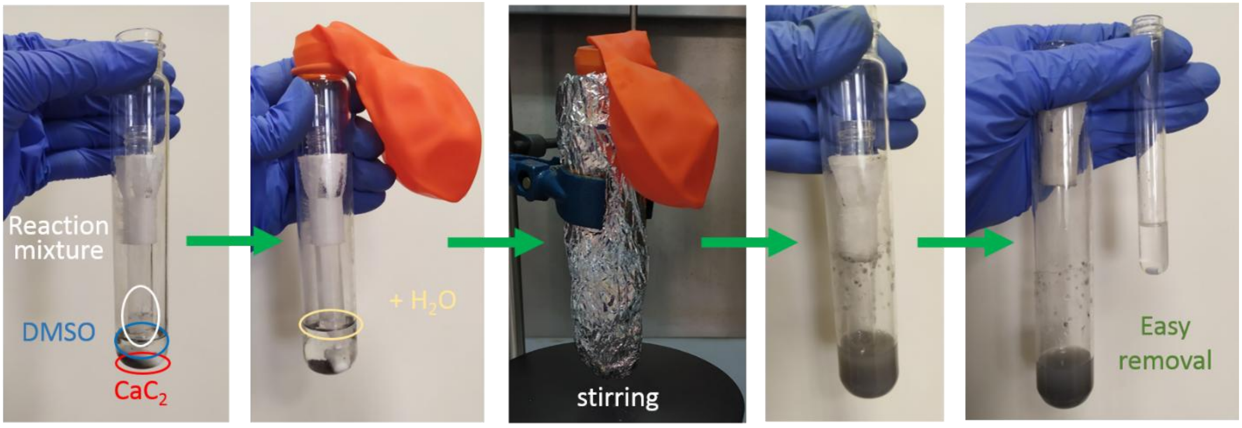

Figure S6. The thiovinylation reaction in a tube-in-tube reactor.

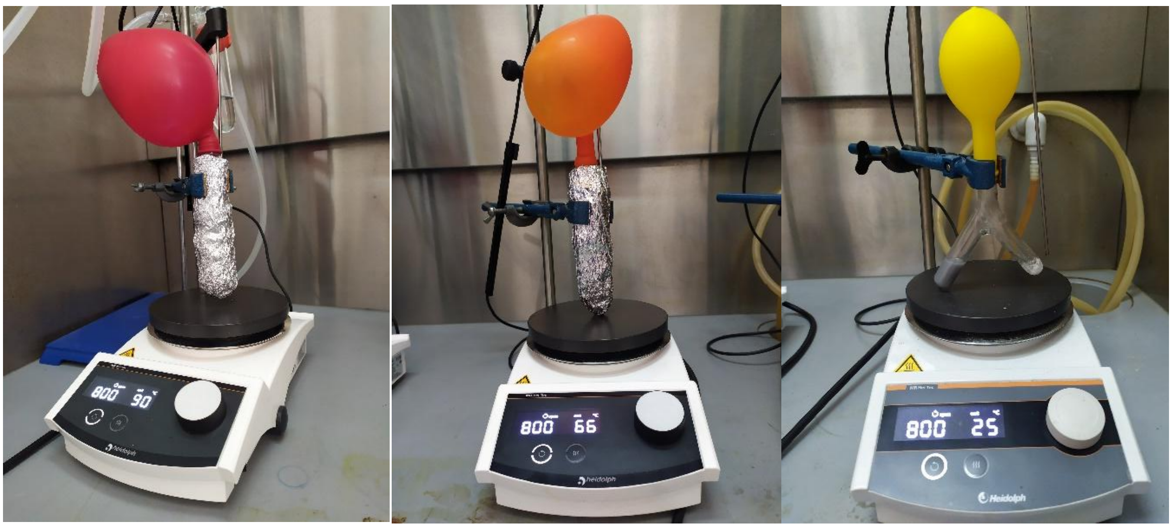

Figure S7: The reaction progress in a tube-in-tube reactor (left and centre) and in a two-chamber reactor (right).

8. Monitoring of the heating with a thermocouple

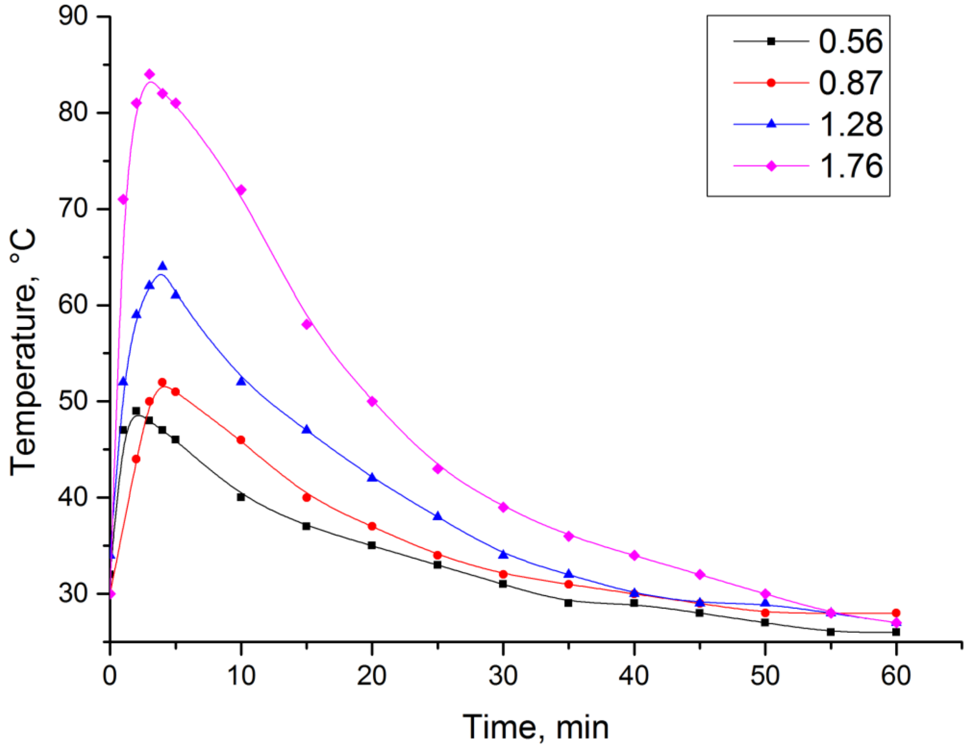

Figure S8: Temperature changes measured with a thermocouple attached to a magnetic stirred. Various loadings of carbide were used: 0.56-1.76 g of carbide.
